# Multi-indication Evidence Synthesis in Oncology Health Technology Assessment: Meta-analysis Methods and Their Application to a Case Study of Bevacizumab

**DOI:** 10.1177/0272989X241295665

**Published:** 2024-11-18

**Authors:** Janharpreet Singh, Sumayya Anwer, Stephen Palmer, Pedro Saramago, Anne Thomas, Sofia Dias, Marta O Soares, Sylwia Bujkiewicz

**Affiliations:** Biostatistics Research Group, Department of Population Health Sciences, University of Leicester, Leicester, UK; Centre for Reviews and Dissemination, University of York, York, UK; Centre for Health Economics, University of York, York, UK; Centre for Health Economics, University of York, York, UK; Leicester Cancer Research Centre, University of Leicester, Leicester, UK; Centre for Reviews and Dissemination, University of York, York, UK; Centre for Health Economics, University of York, York, UK; Biostatistics Research Group, Department of Population Health Sciences, University of Leicester, Leicester, UK

**Keywords:** meta-analysis, health technology assessment, oncology, surrogate endpoints, mixture models, multi-indication drugs

## Abstract

**Background:**

Multi-indication cancer drugs receive licensing extensions to include additional indications, as trial evidence on treatment effectiveness accumulates. We investigate how sharing information across indications can strengthen the inferences supporting health technology assessment (HTA).

**Methods:**

We applied meta-analytic methods to randomized trial data on bevacizumab, to share information across oncology indications on the treatment effect on overall survival (OS) or progression-free survival (PFS) and on the surrogate relationship between effects on PFS and OS. Common or random indication-level parameters were used to facilitate information sharing, and the further flexibility of mixture models was also explored.

**Results:**

Treatment effects on OS lacked precision when pooling data available at present day within each indication separately, particularly for indications with few trials. There was no suggestion of heterogeneity across indications. Sharing information across indications provided more precise estimates of treatment effects and surrogacy parameters, with the strength of sharing depending on the model. When a surrogate relationship was used to predict treatment effects on OS, uncertainty was reduced only when sharing effects on PFS in addition to surrogacy parameters. Corresponding analyses using the earlier, sparser (within and across indications) evidence available for particular HTAs showed that sharing on both surrogacy and PFS effects did not notably reduce uncertainty in OS predictions. Little heterogeneity across indications meant limited added value of the mixture models.

**Conclusions:**

Meta-analysis methods can be usefully applied to share information on treatment effectiveness across indications in an HTA context, to increase the precision of target indication estimates. Sharing on surrogate relationships requires caution, as meaningful precision gains in predictions will likely require a substantial evidence base and clear support for surrogacy from other indications.

**Highlights:**

- We investigated how sharing information across indications can strengthen inferences on the effectiveness of multi-indication treatments in the context of health technology assessment (HTA).
- Multi-indication meta-analysis methods can provide more precise estimates of an effect on a final outcome or of the parameters describing the relationship between effects on a surrogate endpoint and a final outcome.
- Precision of the predicted effect on the final outcome based on an effect on the surrogate endpoint will depend on the precision of the effect on the surrogate endpoint and the strength of evidence of a surrogate relationship across indications.
- Multi-indication meta-analysis methods can be usefully applied to predict an effect on the final outcome, particularly where there is limited evidence in the indication of interest.

## Introduction

In health technology assessment (HTA) of cancer drugs, evidence on a relative treatment effect on overall survival (OS) supports assessments of the clinical and economic value of a drug, relative to a comparator, for a patient population defined by a particular indication (e.g., tumor type). As HTA agencies seek to make a reimbursement decision soon after a drug is licensed, the evidence on treatment effectiveness may be limited and consist only of a single trial, in which the treatment effect is measured on a surrogate endpoint; for example, progression-free survival (PFS) as a surrogate endpoint to OS.^1^ This can result in a large uncertainty in effect estimates on OS, or effect estimates available for PFS alone, at the time the HTA is conducted.

Some cancer drugs are, however, trialled across multiple indications over time. When the trial results in a new indication are reported, the drug’s license may be extended to this new indication.^2^ Despite this accumulation of evidence over time, HTAs typically restrict their scope to consider evidence within a single target indication. However, sharing information across indications could strengthen the evidence base supporting decision making on a new indication. The National Institute for Health and Care Excellence (NICE) in England and Wales has identified the development of evidence synthesis methods for multi-indication HTA of cancer drugs as a key priority.^3^

Explicit evidence synthesis models exist that share information across evidence sets^4^ by defining relationships between parameters corresponding to the different sets of evidence that can be functional, hierarchical, multivariate, or based on a prior distribution in a Bayesian framework. For example, network meta-analysis, which allows for sharing of information across treatment comparisons using functional relationships (i.e., where model parameters are related by deterministic functions), has become an established method to inform treatment effect estimates in HTA.^5^ Multivariate meta-analysis shares information across multiple outcomes and has been proposed for modeling surrogate relationships between treatment effects on a surrogate endpoint (e.g., PFS) and a final clinical outcome (e.g., OS).^3,6,7^

Sharing of information has been previously applied in the multi-indication context. Panoramic meta-analysis^8,9^ and models for the analysis of basket trials of histology-independent oncology drugs^10,11^ have used hierarchical models where indication-level parameters are assumed to vary according to a common distribution (full exchangeability). In these models, the data determine the strength of sharing. However, more flexible models that do not presume full exchangeability and avoid sharing information from extreme indications would provide more robust inferences and may be more suitable to support decision making. In addition, the possibility of incorporating clinical opinion to regulate the degree of sharing where there is uncertainty regarding the plausibility of sharing would be valuable.

To allow for added flexibility in sharing assumptions, mixture models can be considered. The simplest mixture model consists of a weighted mixture of 2 components: an informative prior distribution and a vague prior distribution.^12^ The informative prior distribution represents the sharing component with parameter values based on data from the other strata. The vague prior distribution provides robustness in strata where there is uncertainty regarding whether sharing is appropriate. Extensions include models where the sharing component consists of a common parameter across strata^13^ or a hierarchical relationship across strata.^14^ Mixture models have been applied in other contexts of evidence synthesis; for example, Papanikos et al.^15^ shared information on parameters of surrogate relationships across treatment classes but have not been evaluated for use in the multi-indication context.

In this article, we demonstrate the application of mixture models to multi-indication meta-analysis in oncology to enable sharing of information on individual endpoints and on a surrogate relationship, where a predicted treatment effect on OS for the target indication is the key estimate of interest. We use the example of bevacizumab, one of the earliest multi-indication oncology drugs, which has been trialled across multiple cancer indications and been the subject of several NICE technology appraisals (TAs).

## Case Study: Bevacizumab

Bevacizumab is an angiogenesis inhibitor used as a targeted therapy for different solid tumors.^2^ It has received multiple licensing extensions and is currently approved in the United Kingdom as a treatment for 7 cancer indications, where it has been the subject of 11 NICE TAs.

A literature search was conducted to identify randomized controlled trial (RCT) evidence across bevacizumab’s licensed indications in the advanced or metastatic cancer setting: breast cancer (BC), cervical cancer (CC), colorectal cancer (CRC), glioblastoma (GBM), non-small-cell lung cancer (NSCLC), ovarian fallopian tube, primary peritoneal (OFTPP) cancer, and renal cell carcinoma (RCC).

A total of 41 RCTs were identified. Most trials compared the addition of bevacizumab to background therapy, typically chemotherapy. All 41 trials reported effect estimates for PFS, and 36 reported effect estimates for OS. The accumulation of evidence on the effectiveness of bevacizumab over time in the context of NICE TAs is described in Appendix A in Supplemental material.

**Figure 1.**
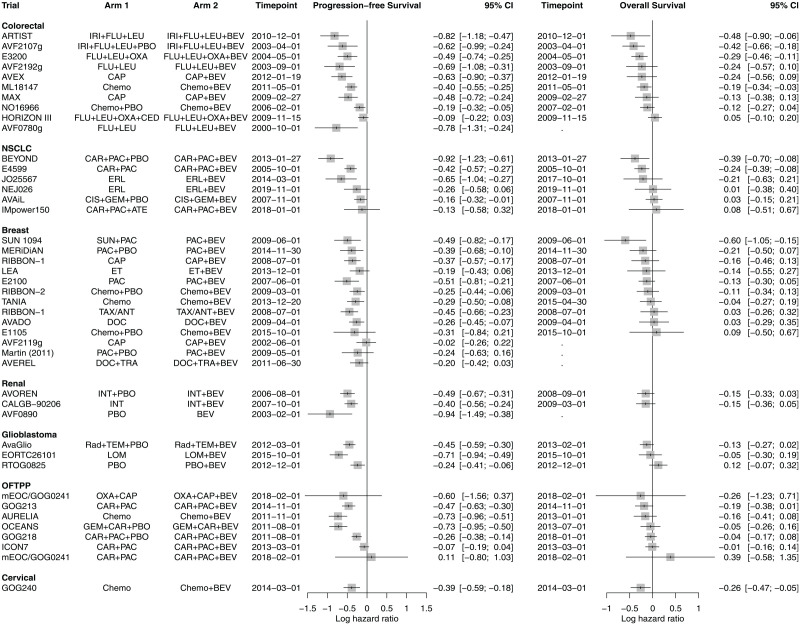
Forest plot summarizing log-transformed hazard ratio estimates on progression-free survival (PFS) and overall survival (OS) from randomized controlled trials assessing bevacizumab across cancer indications. Estimates are ordered by OS effect size within each cancer indication. BEV, bevacizumab; NSCLC, non-small-cell lung cancer; OFTPP, Ovarian, fallopian tube and primary peritoneal. A list of full treatment names is provided in Appendix B.1 in Supplemental material.

Figure 1 presents the final log hazard ratio (HR) estimates from each trial, for PFS (left) and OS (right). The estimates are ordered by OS effect size (largest to smallest) within each indication. Most trials showed a significantly favorable effect of bevacizumab on PFS, and this is consistent both within and across indications. There is more uncertainty around effect estimates on OS, where point estimates indicate a favorable effect of bevacizumab but statistical significance is some achieved in only 7 trials. For the indications with relatively more trials (CRC, NSCLC, and OFTPP), there is some evidence of between-studies heterogeneity in PFS estimates (demonstrated by nonoverlapping confidence intervals), although this is more difficult to establish on OS. The data show that larger effect estimates on PFS correspond to larger effect estimates on OS, particularly for CRC, NSCLC, and OFTPP cancer, which is suggestive of a potential surrogate relationship between these outcomes.

## Methods

In this section, we define the models and detail their implementation. All models are defined in a Bayesian framework. We describe the models in 2 parts. The first part relates to the within-indication model component that synthesizes treatment effects on OS (univariate models) or evidence for surrogate relationships between treatment effects on PFS and OS (bivariate models). Both approaches synthesize data on the log HR scale. The second part describes the between-indications component of the models. We consider 5 alternative approaches to relate the indication-specific parameters, that facilitate different levels of sharing of information across indications. We apply each of the 5 sharing approaches to both the univariate and bivariate models.

### Models for Multi-indication Meta-analysis

#### Within-indication component: within-study and between-studies models

Modeling approaches considered at the within-indication level use standard meta-analytic specifications for the within-study (likelihood) and the between-studies models.

The univariate models use the normal-normal hierarchical (random effects) model,^16^ which assumes that the relative treatment effects, $Y_{\mathrm{ij}}$ (in study i within indication j ), are normally distributed, with associated standard errors $\sigma_{\mathrm{ij}}^{2}$ (assumed known) and mean defined by the true treatment effects $\delta_{\mathrm{ij}}$ ,

(1) $$Y_{ij}\sim N\left(\delta_{ij},\sigma_{ij}^{2}\right).$$

At the between-studies level, the true treatment effects are assumed to be exchangeable across studies within each indication,

(2) $$\delta_{ij}\sim N\left(d_{j},\tau_{j}^{2}\right),$$

where $d_{j}$ is the pooled effect for indication j and $\tau_{j}$ is the associated (within-indication) between-studies standard deviation. A weakly informative half-normal prior distribution is placed on the between-studies standard deviation parameters, $\tau_{j}\sim \left|N\left(0,0.5^{2}\right)\right|$ , as recommended by Röver et al.,^17^ which are assumed to be independent across indications in all analyses. For the purpose of multi-indication meta-analysis, we seek to share information on the pooled effects, $d_{j}$ .

The bivariate modeling approach uses the formulation by Daniels and Hughes^7^ to examine a linear surrogate relationship between the treatment effects on the surrogate endpoint and on the final clinical outcome within each indication. For each study i and indication j , data on the effects are available (e.g., as log HR estimates) on a surrogate endpoint, $Y_{1\mathrm{ij}}$ , and a final outcome, $Y_{2\mathrm{ij}}$ , with associated standard errors $\sigma_{1\mathrm{ij}}^{2}$ and $\sigma_{2\mathrm{ij}}^{2}$ , respectively, and within-study correlation $\rho_{\mathrm{wij}}$ . At the within-study level, the correlated observed effects are assumed to follow a bivariate normal distribution,

(3) $$\left(\begin{array}{c} Y_{1ij}\\ Y_{2ij} \end{array}\right)\sim N(\left(\begin{array}{c} \delta_{1ij}\\ \delta_{2ij} \end{array}\right),(\begin{array}{cc} \sigma_{1ij}^{2} & \sigma_{1ij}\sigma_{2ij}\rho_{wij}\\ \sigma_{1ij}\sigma_{2ij}\rho_{wij} & \sigma_{2ij}^{2} \end{array}))$$

where $\delta_{1ij}$ and $\delta_{2\mathrm{ij}}$ are the correlated true effects on the surrogate endpoint and final clinical outcome, respectively. The within-study correlation between the effects on the 2 outcomes is rarely reported but can be informed by external data or assigned a prior distribution.^6^ We use a vague uniform prior distribution in our implementation $\rho_{\mathrm{wij}}\sim U\left(-1,1\right)$ .

At the between-studies level, for each indication j , the true effects on the final outcome, $\delta_{2\mathrm{ij}}$ , are assumed to have a linear relationship with the true effects on the surrogate endpoint $\delta_{1\mathrm{ij}}$ ,

(4) $$\delta_{2ij}\sim N\left(\lambda_{0j}+\lambda_{1j}\delta_{1ij},\psi_{j}^{2}\right)$$

where $\lambda_{0j}$ and $\lambda_{1j}$ represent the intercept and slope, respectively, and $\psi_{j}^{2}$ is the variance of the true effects on the final outcome conditional on the true effects on the surrogate endpoint (conditional variance) within indication j .

These parameters can be used to assess the strength of the surrogate relationship using the criteria proposed by Daniels and Hughes.^7^ Where a null effect on the surrogate endpoint should imply a null effect on the final outcome ( $\lambda_{0j}=0$ ), the slope should indicate an association between the endpoints ( $\lambda_{1j}\neq 0$ ), and the conditional variance measures to what extent the treatment effect on the final outcome can be predicted from the effect on the surrogate endpoint (with $\psi_{j}^{2}=0$ corresponding to perfect predictions). Vague normal prior distributions are placed on the true effects corresponding to the surrogate endpoint, $\delta_{1\mathrm{ij}}\sim N\left(0,10^{2}\right)$ , which are assumed to be independent across studies. For the purpose of multi-indication meta-analysis, we seek to share information on the intercept ( $\lambda_{0j}$ ), slope ( $\lambda_{1j}$ ), and conditional variance parameters ( $\psi_{j}^{2}$ ), across indications.

#### Between-indications component

We apply 5 distinct between-indications models implying different degrees of sharing of information across indications. We denote the indication-level parameters by the vector $\theta_{j}$ . In the bivariate approach, the 5 models share information for each of the 3 surrogacy parameters (intercept, slope, and conditional variance), but the sharing relationships are implemented independently for each parameter and therefore the level of sharing could differ between the parameters.

##### Independent parameters (IP) model

Here, independent prior distributions are placed on the $\theta_{j}$ ,

(5) $$\theta_{j}\sim P().$$

We use a vague normal prior distribution for the univariate approach, $P(d_{j})=N(0,10^{2})$ , and we use the following vague prior distributions for the bivariate approach; $P(\lambda_{0j})=N(0,10^{2})$ , $P(\lambda_{1j})=N(0,10^{2})$ , and $P(\psi_{j})=|N(0,0.5^{2})|$ .

In the IP model, the indication-level parameters are informed by direct (within-indication) evidence only, and there is no sharing of information across indications. This model provides a reference to compare the treatment effect estimates from other models where sharing is allowed.

##### Common parameter (CP) model

Here, we assume a common overall vector of parameters, θ , which pools the indication-specific parameter values,

(6) $$\theta_{j}=\theta .$$

We use a vague normal prior distribution for the univariate approach, $P(d)=N(0,10^{2})$ , and the following vague prior distributions for the bivariate approach: $P(\lambda_{0})=N(0,10^{2})$ , $P(\lambda_{1})=N(0,10^{2})$ , and $P(\psi )=|N(0,0.5^{2})|$ .

The CP model implements the maximum sharing of information, serving as a polar opposite reference to the IP model, enabling an assessment of the assumption of equal effects between indications.

##### Mixed common and independent parameters (MCIP) model

Here, a mixture of common and independent parameters (MCIP) is assumed. This is facilitated by a mixture indicator variable, $c_{j}$ , which follows a Bernoulli distribution. Where this variable equals 1, the $\theta_{j}$ are assumed equivalent, where it is equal to 0, $\theta_{j}$ are assumed independent,

(7) $$\begin{array}{l} \theta_{j}=\{\begin{array}{cc} \theta , & c_{j}=1\\ \theta_{j}\sim P(), & c_{j}=0 \end{array}\\ c_{j}\sim Bernoulli(p_{j}). \end{array}$$

The mixture hyperparameter, $p_{j}$ , is assigned a vague beta prior distribution, $p_{j}\sim \beta (1,1)$ , to reflect uncertainty in its values. The posterior mean of the mixture indicator, $c_{j}$ , represents the mixture probability quantifying the level of mixing.

The MCIP model allows the data to determine, for each indication, the plausibility of the IP and CP assumptions. Note that, in this model, the overall pooled parameter, θ , does not represent a common effect across all indications in the data set but rather a common effect conditional on such a common effect existing. For example, an indication with data that is extreme in relation to those of other indications is expected to be estimated to have a small mixture probability value and, for this reason, is expected to make only a negligible contribution to the overall pooled parameter estimate.

##### Random parameters (RP) model

Here, the indication-level parameters are assumed to be fully exchangeable (also termed *random*) and vary according to a common distribution,

(8) $$\theta_{j}|\eta_{j}\sim F(\eta_{j}).$$

The exchangeability distribution is defined as normal. For the univariate approach, it is defined as

(9) $$d_{j}|m_{d},\tau_{d}\sim F(m_{d},\tau_{d})=N(m_{d},\tau_{d}^{2}),$$

where $m_{d}$ is the overall pooled effect and $\tau_{d}$ is the between-indications standard deviation. A vague normal prior distribution is placed on the pooled parameter $m_{d}\sim N(0,10^{2})$ , and a weakly informative half-normal prior distribution is placed on the standard deviation parameter $\tau_{d}\sim |N(0,0.5^{2})|$ .

For the bivariate between-indications model, the exchangeability distributions are given by

(10) $$\begin{array}{l} \lambda_{0j}|\beta_{0},\xi_{0}\sim F(\beta_{0},\xi_{0})=N(\beta_{0},\xi_{0}^{2})\\ \lambda_{1j}|\beta_{1},\xi_{1}\sim F(\beta_{1},\xi_{1})=N(\beta_{1},\xi_{1}^{2})\\ \psi_{j}|h\sim F(h)=|N(0,h)| \end{array}$$

where $\beta_{0}$ and $\beta_{1}$ are the overall pooled intercept and slope parameters, respectively; $\xi_{0}$ and $\xi_{1}$ are the associated between-indications standard deviation parameters; and h is the between-indications variance of the conditional variances. Prior distributions assigned are $\beta_{0},\beta_{1}\sim N(0,10^{2})$ , $\xi_{0},\xi_{1}\sim |N(0,0.5^{2})|$ , and h~Γ(1,0.01) .

The RP model assumes full exchangeability, with the data determining the level of sharing via the parameters of the common distribution. The between-indications standard deviation parameters quantify the level of heterogeneity between indications. Smaller standard deviation values suggest that the effect estimates are expected to be more similar across indications (with results of the RP model approximating those of the CP model), and larger values suggest that the effect estimates differ significantly from each other (with results of the RP model approximating those of the IP model). Within this model, indication-specific effects are shrunk toward the overall mean effect. The degree of shrinkage depends on the data. An extreme but imprecise IP model effect is likely to be significantly shrunken in the RP model toward the overall mean while retaining large uncertainty. Indication-level estimates that are less extreme and imprecise may become more precise but with the point estimate not significantly changed.

##### Mixed random and independent parameters (MRIP) model

Here, a mixture of random and independent parameters (MRIP) is assumed:

(11) $$\begin{array}{l} \theta_{j}\sim \{\begin{array}{cc} F(\eta_{j}), & c_{j}=1\\ P(), & c_{j}=0 \end{array}\\ c_{j}\sim Bernoulli(p_{j}) \end{array}$$

where $c_{j}$ and $p_{j}$ are the mixture indicator and mixture hyperparameter variables, respectively, and have the same interpretation as in the MCIP model. Here, $F(\eta_{j})$ represents the exchangeability distribution that is the same as in the RP model (equations 9 and 10), and P() is the set of vague prior distributions that are the same as those defined for the IP model.

The MRIP model provides additional flexibility in relation to the RP model for the data to determine, for each indication, the plausibility of the RP assumption via the mixture indicator. In a similar way to the MCIP model, the parameters describing the exchangeability distribution in the MRIP model represent a common distribution across indications where a common distribution is deemed to exist (as determined by the data).

Within this model, $\theta_{j}$ represents the indication-specific estimate that is a weighted-average (i.e., weighted by the mixture probability) of the exchangeable and independent components. For the univariate model, $d_{j}$ is the indication-specific treatment effect estimate that is informed by sharing information across indications (via the exchangeability distribution), where the degree of sharing is regulated by the mixture probability.

#### Obtaining estimates of a treatment effect on OS

This section describes how to obtain predictions for an effect on OS from each of the univariate sharing models by including a parameter representing the predicted effect for a new indication. For the hierarchical models (RP and MRIP), the effect for a new indication can be sampled from the predictive distribution given by $d_{\mathrm{pred}}\sim N(m_{d},\tau_{d}^{2})$ . In the HTA context, policy decisions are made for a population, and the estimates used to inform decision-making must take heterogeneity in the treatment effects into consideration. As such, the mean of the common distribution in the hierarchical models could lead to overprecise estimates of the treatment effect, and the predictive distribution is recommended as a basis for decision making.^18^

For the bivariate models, predictions of the average treatment effect on OS in a target indication can be obtained by using indication-level parameter estimates obtained from the between-indications component of the above models for sharing of information on the intercept ( $\lambda_{0j}$ ), slope ( $\lambda_{1j}$ ), and conditional variance ( $\psi_{j}^{2}$ ) parameters (see equation (4) in the “Within-Indication Component: Within-Study and Between-Studies Models” section).

Consequently, the surrogate relationship estimated by the bivariate model needs to be applied to an indication-specific average effect estimate for PFS to predict an effect on OS. Decisions over the sharing of information on this indication-specific PFS effect estimate can differ from those on the bivariate model into which it is entered. We implemented 2 options. The first enters the indication-level PFS estimate from the univariate IP model into the indication-specific surrogate relationships estimated by each of the bivariate sharing models (CP, MCIP, RP, MRIP). In this case, the predicted effect on OS will be based on sharing information on the surrogate relationships but not on the effects on PFS across indications. The second approach involves matching the between-indications relationship to share on both effects on PFS and surrogacy parameters (i.e., the PFS effect estimate from the CP univariate model is entered into the surrogate relationship estimated by the CP bivariate model, etc.). Formulae for obtaining a predicted treatment effect on OS from an effect estimate on PFS are described in Appendix C in Supplemental material.

### Model Implementation

#### Application to case study

The proposed methods will be initially applied to all available bevacizumab data (present-day analysis, see Figure 1), across all indications. Given that bevacizumab was one of the earliest multi-indication oncology drugs, this analysis will examine a well-developed evidence base. Results from the present-day analysis will represent a benchmark with which to compare the results from the more policy-relevant analyses at (earlier) appraisal time points, where less evidence had accumulated across indications. The analyses at 2 particular appraisal time points (TA178^27^ in RCC and TA285^28^ in OFTPP cancer) assume we want to estimate effects for the target indication in those appraisals using the multi-indication data available at the time of each appraisal (see figures in Appendix D in Supplemental material for a summary of each data set).

#### Software, code, and model implementation

All models were implemented in OpenBUGS, via the *R2OpenBUGS* package (version 3.2.1)^21^ in R (version 4.1.3),^22^ using MCMC sampling to estimate Bayesian posterior distributions for model parameters. Each implementation consisted of 3 MCMC chains, where 20,000 iterations were used as an initial burn-in period for each chain. Posterior estimates were based on 80,000 samples per chain and checked for sensitivity to changes in initial values. The effective sample size and $\hat{R}$ statistics were used to assess nonconvergence of chains.^23^ Example BUGS code to implement the multi-indication meta-analysis models is publicly available on GitHub.^24^

#### Assessment of fit and model choice

The deviance information criterion (DIC) provides a measure of how well a model fits a data set while penalizing model complexity in terms of the number of parameters, where lower values are preferred.^25^ In our application, we use the residual deviance and DIC to compare multi-indication univariate and bivariate models (IP, CP, MCIP, RP, MRIP) separately, at each analysis time point. We use a difference of 3 units in DIC to represent a meaningful difference in model fit between 2 models, as recommended by Spiegelhalter et al.^25^

## Results

We report the results from applying the multi-indication meta-analysis methods described in the “Methods” section to the data available at the following 3 time points (see Appendix Figure A1 in Supplemental material for an illustration of trial data with respect to these time points): present day, TA178 (RCC), and TA285 (OFTPP cancer). We first consider methods for sharing of information on treatment effects on an individual endpoint then methods for sharing on a surrogate relationship between effects on 2 endpoints.

### Present-Day Analyses

**Figure 2:**
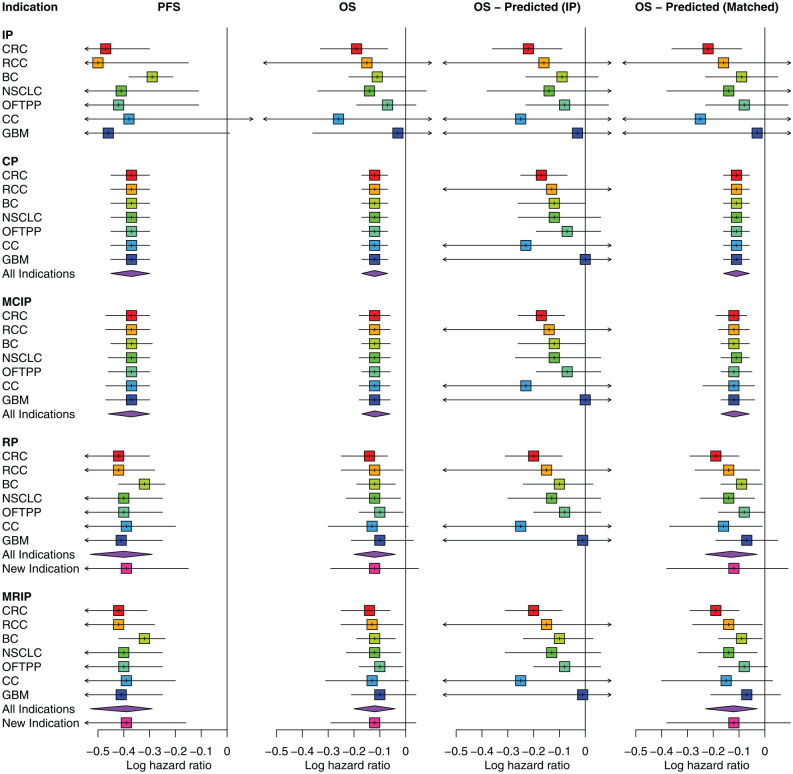
Treatment effect estimates (on the log hazard ratio scale) from multi-indication meta-analysis of data available at present day. For the CP model, the indication-level estimates are all equivalent to the “All indications” estimate on the PFS, OS, and OS - Predicted (Matched) outcomes. BC, breast cancer; CC, cervical cancer; CP, common parameter; CRC, colorectal cancer; GBM, glioblastoma; IP, independent parameters; MCIP, mixed common and independent parameters; MRIP, mixed random and independent parameters; NSCLC, non-small-cell lung cancer; OFTPP, ovarian, fallopian tube, and primary peritoneal; OS, overall survival; PFS, progression-free survival; RCC, renal cell carcinoma; RP, random parameters.

Figure 2 illustrates the results of the analyses of data available at present day. It presents the median and 95% credible interval (CrI) for the treatment effect estimates corresponding to each indication, grouped by the between-indications model. The columns denoted as “PFS” and “OS” include the estimates from the application of the univariate models to synthesize effects on PFS and OS, respectively. The predicted effects on OS obtained from the application of the bivariate models are included in the columns identified as “OS - Predicted (IP)” and “OS - Predicted (Matched).” We will describe the results from the univariate and bivariate models in turn.

#### Results from univariate analyses of treatment effects on individual endpoints

In line with the trial-level results (presented in Figure 1), univariate synthesis of treatment effects on PFS suggests bevacizumab to be beneficial across the majority of indications. There is large overlap in the CrIs for the indication-level effect estimates from the IP model and little difference between the effect estimates by the MCIP and CP models. Together, these results represent limited heterogeneity in the effects across indications. Where within-indication evidence (IP model) is insufficient for the treatment effect estimates to be statistically meaningful (e.g., for CC and GBM), sharing of information across all indications increases the precision of the estimates leading to CrIs that do not include the null effect (CP, MCIP, RP, and MRIP models).

A positive treatment effect on OS is also apparent from within-indication evidence (IP model) for those indications with more data, for example, CRC (9 trials) and BC (10 trials), but data are too limited to make conclusions for the other indications. Thus, there is no suggestion from these data that sharing on OS is inappropriate.

Estimates from the CP model, which assumes equality of the treatment effects and provides maximal sharing across indications, suggest that a meaningful positive effect can be expected on both outcomes. The RP model, which allows for variability in effects across indications, provides indication-specific estimates that are more similar to each other compared with those from the IP model (i.e., they are shrunken toward the overall mean) but retain wider uncertainty for the indications with sparser data (e.g., CC or GBM). Notably, the point estimate of the PFS effect for BC has not been substantially shrunken (due to the higher level of evidence for this indication and discrepancy with the point estimates from the other indications).

The estimates obtained from the mixture models are in close agreement with those from the corresponding nonmixture sharing model (i.e., MCIP with CP, MRIP with RP), and the mixture probabilities are approximately equal to 1 for most indications (see Table 1 for the mixture probability estimates). These results add plausibility for sharing across indications in this case study as the data are relatively consistent. The discrepancy in the point estimate for the PFS effect in BC is accounted for by the MCIP model, by attributing this indication a lower mixture probability (mean 0.96, standard deviation 0.19) compared with the other indications (see Table 1). There is no meaningful difference in the goodness-of-fit between the different models for the synthesis of treatment effects on both OS and PFS, as the DIC values (presented in Appendix E.1.3 in Supplemental material) differ by less than 3 units. This suggests that although any model could be considered appropriate, the application of the simpler CP model is most efficient in describing these data.

**Table 1:** Mixture Probability Estimates (as Mean and Standard Deviation) and Informative and Vague Mixture Component Estimates (as Median and 95% Credible Interval) from Application of Models to Data on Overall Survival (OS) and Progression-Free Survival (PFS) at Present Day

Model	Indication	Probability	Informative	Vague
OS				
MCIP	BC	1.00 (0.05)	−0.12 (−0.17, −0.06)	−0.10 (−61.80, 61.70)
MCIP	CC	0.99 (0.11)	−0.12 (−0.17, −0.06)	−0.21 (−61.70, 61.85)
MCIP	CRC	0.99 (0.08)	−0.12 (−0.17, −0.06)	−0.14 (−62.23, 61.94)
MCIP	GBM	0.99 (0.08)	−0.12 (−0.17, −0.06)	0.02 (−62.00, 61.71)
MCIP	NSCLC	1.00 (0.06)	−0.12 (−0.17, −0.06)	−0.13 (−61.95, 62.01)
MCIP	OFTPP	1.00 (0.07)	−0.12 (−0.17, −0.06)	−0.04 (−61.79, 61.94)
MCIP	RCC	1.00 (0.07)	−0.12 (−0.17, −0.06)	−0.07 (−61.84, 61.94)
MRIP	BC	1.00 (0.07)	−0.12 (−0.19, −0.04)	−0.06 (−61.65, 61.84)
MRIP	CC	0.99 (0.11)	−0.13 (−0.30, 0.01)	−0.11 (−61.83, 61.82)
MRIP	CRC	0.99 (0.07)	−0.14 (−0.25, −0.06)	−0.18 (−61.92, 61.69)
MRIP	GBM	0.99 (0.08)	−0.10 (−0.21, 0.03)	−0.02 (−61.72, 61.67)
MRIP	NSCLC	1.00 (0.06)	−0.12 (−0.23, −0.02)	−0.07 (−62.01, 62.20)
MRIP	OFTPP	1.00 (0.06)	−0.10 (−0.18, −0.01)	−0.08 (−61.97, 61.95)
MRIP	RCC	0.99 (0.07)	−0.13 (−0.25, −0.01)	−0.08 (−61.95, 61.64)
PFS				
MCIP	BC	0.96 (0.19)	−0.37 (−0.46, −0.30)	−0.29 (−61.28, 61.39)
MCIP	CC	0.99 (0.09)	−0.37 (−0.46, −0.30)	−0.16 (−61.79, 61.67)
MCIP	CRC	0.99 (0.08)	−0.37 (−0.46, −0.30)	−0.34 (−61.80, 61.82)
MCIP	GBM	0.99 (0.08)	−0.37 (−0.46, −0.30)	−0.30 (−62.12, 61.94)
MCIP	NSCLC	1.00 (0.06)	−0.37 (−0.46, −0.30)	−0.21 (−61.69, 62.19)
MCIP	OFTPP	1.00 (0.06)	−0.37 (−0.46, −0.30)	−0.01 (−62.14, 62.23)
MCIP	RCC	0.99 (0.10)	−0.37 (−0.46, −0.30)	−0.29 (−61.95, 61.70)
MRIP	BC	0.99 (0.11)	−0.32 (−0.43, −0.24)	−0.28 (−61.82, 61.36)
MRIP	CC	0.99 (0.10)	−0.39 (−0.61, −0.20)	−0.19 (−61.71, 61.70)
MRIP	CRC	0.99 (0.08)	−0.42 (−0.58, −0.30)	−0.36 (−61.73, 62.01)
MRIP	GBM	0.99 (0.09)	−0.41 (−0.60, −0.25)	−0.14 (−61.79, 62.11)
MRIP	NSCLC	0.99 (0.08)	−0.40 (−0.57, −0.25)	−0.28 (−61.94, 61.63)
MRIP	OFTPP	0.99 (0.07)	−0.40 (−0.57, −0.25)	−0.17 (−61.67, 62.03)
MRIP	RCC	0.99 (0.09)	−0.42 (−0.61, −0.28)	−0.31 (−61.39, 62.16)

BC, breast cancer; CC, cervical cancer; CRC, colorectal cancer; GBM, glioblastoma; MCIP, mixed common and independent parameters; MRIP, mixed random and independent parameters; NSCLC, non-small-cell lung cancer; OFTPP, ovarian, fallopian tube, and primary peritoneal; RCC, renal cell carcinoma.

#### Results of surrogate endpoint evaluation

**Figure 3:**
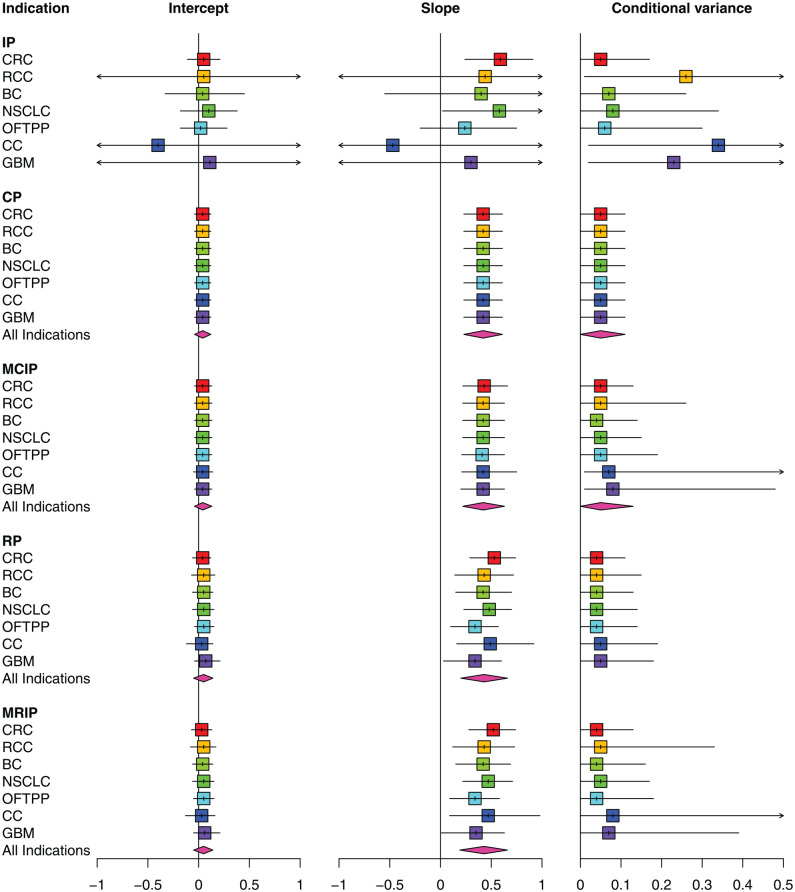
Multi-indication meta-analysis of surrogacy parameters using the data available at present day. For the CP model, the indication-level estimates are all equivalent to the “All indications” estimate. BC, breast cancer; CC, cervical cancer; CP, common parameter; CRC, colorectal cancer; GBM, glioblastoma; IP, independent parameters; MCIP, mixed common and independent parameters; MRIP, mixed random and independent parameters; NSCLC, non-small-cell lung cancer; OFTPP, ovarian, fallopian tube, and primary peritoneal; RCC, renal cell carcinoma; RP, random parameters.

Before interpreting the results from using the bivariate models to predict a treatment effect on OS in the “Predicted Effects on OS Using Surrogate Relationships” section, we focus on the inferences obtained for the parameters describing the surrogate relationship (i.e., intercept, slope, and conditional variance parameters) between treatments effects on PFS and OS. Figure 3 depicts the median and 95% CrI estimates for these parameters based on the present-day data set, where each column corresponds to a particular parameter and each row includes the indication-specific estimate. The estimates are grouped by the between-indications model. Further results of this analysis can be found in Appendix E.1.4, and Appendix E.4 in Supplemental material presents the results from a cross-validation procedure used to evaluate the surrogate relationship in more detail.^6^ Note that the intercept parameter is estimated on the same scale as a treatment effect on the final outcome (OS) and can be interpreted as a log HR. The slope parameter quantifies how the effect on the final outcome, OS, varies with respect to the effect on the surrogate endpoint, PFS, and as such can be interpreted as a ratio of log HRs.

##### IP model: no sharing across indications

The results from applying the IP model show that the surrogacy parameters are estimated with large uncertainty in indications with limited data: RCC (2 trials), CC (1 trial): and GBM (3 trials). Consequently, it is difficult to determine whether the surrogacy criteria (described in the “Within-Indication Component: Within-Study and Between-Studies Models” section) are satisfied for these indications by considering the within-indication evidence alone. Across all indications, CrIs for intercepts contain the value zero (as desired according to the surrogacy criteria). The CrIs for the slope parameter for CRC and NSCLC (indications with data on both PFS and OS effects from 6,067 patients across 9 trials and 3,034 patients across 6 trials, respectively) do not include zero. This suggests a positive association between the treatment effects on the 2 outcomes within these indications, in which a larger effect on PFS is associated with a larger effect in OS and is indicative of a strong surrogate relationship. However, the CrIs corresponding to the slope parameter for BC and OFTPP cancer include zero, despite the availability of a similar level of data (from 4,459 patients across 10 trials in BC and 4,345 patients across 7 trials in OFTPP cancer). This weak evidence of surrogate relationships encourages caution in the interpretation of the results from the less flexible sharing models, particularly those assuming common or exchangeable surrogacy parameters across all indications (CP and RP).

##### Conditional variance

The conditional variance estimates were relatively large for RCC, CC, and GBM (compared with other indications), reflecting the sparse evidence for determining surrogacy in these indications. Of the remaining indications (BC, NSCLC, OFTPP cancer, and CRC), CRC presents the smallest conditional variance estimate with the narrowest CrI, due to the higher level of evidence for the existence of a surrogate relationship (with precise estimates for all 3 surrogacy parameters). The higher uncertainty in NSCLC and OFTPP cancer estimates (in relation to CRC) may be due to the relatively small number of studies—6 and 7 studies, respectively. In contrast, the higher uncertainty in BC estimates (in relation to CRC), in which the number of studies was 10, is indicative of a weaker surrogate relationship within this indication. The point estimates of the conditional variance parameter corresponding to BC, NSCLC, and OFTPP cancer all appear to be larger compared with the point estimate for CRC, but this may be due to the higher uncertainty in these indications and a positively skewed distribution for this parameter.

##### Models allowing for sharing across indications

Indication-specific estimates from the sharing models are more precise compared with those from the IP model. For instance, the slope parameter estimates (including CrIs) are positive across indications for all sharing models. An exception to this are the conditional variance estimates from applying the mixture models (MCIP and MRIP), which still show relatively large uncertainty for the indications with sparser data: RCC, CC, and GBM. Mixture probabilities for the intercept and slope parameters are all approximately equal to 1 (see Appendix E.1.6 in Supplemental material) but range between 0.57 and 0.82 for the conditional variance parameter, assuming lowest values in CC and GBM.

##### Assessment of model fit

In terms of goodness of fit (see Appendix E.1.5 in Supplemental material), the mixture models have notably smaller DIC values compared with the nonmixture models, with the lowest DIC value for the MRIP model. In this application, the added flexibility of the mixture models (i.e., the ability to regulate the degree of sharing for each indication) is likely to be more suitable for sharing on the conditional variance parameter as the corresponding mixture probabilities are lower and more variable. This indicates a lack of support from the data for the assumptions of equivalence or exchangeability across indications on this parameter.

#### Predicted effects on OS using surrogate relationships

The predicted treatment effects on OS obtained from the bivariate models for each indication are depicted in Figure 2, in columns “OS - Predicted (IP)” and “OS - Predicted (Matched)” (see the “Obtaining Estimates of a Treatment Effect on OS” section for a description of how these are obtained).

The predicted OS effects estimated by the bivariate IP model are consistent with the pooled OS effects estimated by the univariate IP model, albeit with slightly larger uncertainty (see Table E3 in Appendix E.1.2 in Supplemental material for specific numerical values). The predicted OS effects estimated by the bivariate models that allow for sharing of information on the surrogacy parameters (but without sharing on the PFS effect) are only slightly more precise compared with estimates from the bivariate IP model, even when implementing a CP assumption. This demonstrates the limited impact of sharing on the surrogate relationship alone (when not sharing on the PFS effect). It is only when sharing on both the PFS effect and the surrogate relationship (OS - Predicted [Matched] column) that notable precision gains are obtained in relation to not sharing. This suggests that sharing on the PFS effect, to obtain a more precise pooled estimate, is particularly important in predicting the corresponding OS effect using the bivariate models.

Considering the matched bivariate models, which allow for sharing on both the PFS effect and the surrogate relationship (by matching the sharing assumption made on the 2 sets of parameters), the predicted estimates from the bivariate CP model show the lowest uncertainty, which is to be expected as this model allows maximal sharing. These estimates are consistent with the pooled OS estimates from the univariate CP model. The predicted estimates from the bivariate MCIP model show wider uncertainty than CP model estimates do. The bivariate RP and MRIP models present more variation in the point estimates across all indications, all being in line with the IP model but shrunken toward the overall mean value. The shrinkage is greater for indications other than CRC and BC.

The level of uncertainty in the OS effect estimates predicted by the matched bivariate sharing models is similar to the uncertainty in the pooled OS effects estimated by the univariate sharing models. However, there are some differences in the estimates between the univariate and bivariate approaches. For instance, the bivariate approach leads to higher variation in point estimates across indications. Furthermore, predictions for a new indication made by the hierarchical sharing models (RP and MRIP) have larger uncertainty in the bivariate context. For CC, for which data were available from only a single trial, the RP model CrIs include the null effect when sharing across indications on OS alone. However, this is not the case when predicting the OS effect by sharing on both the PFS effect and the surrogacy parameters (see CrI in OS - Predicted [Matched] column).

### Appraisal Specific Analyses

**Figure 4:**
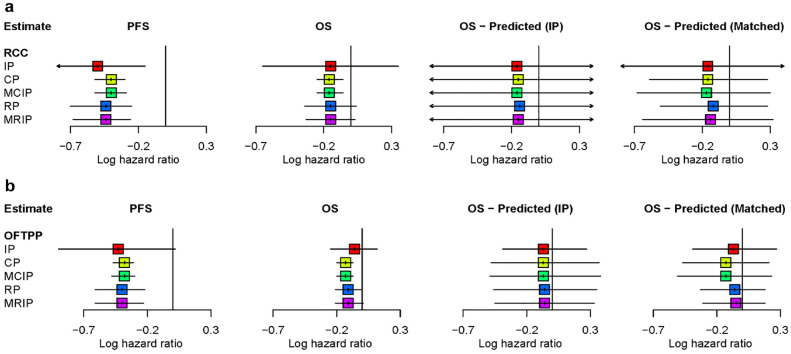
Multi-indication meta-analysis of data available at the time of (a) TA178 (target indication: renal cell carcinoma [RCC]) and (b) TA285 (target indication: ovarian, fallopian tube, and primary peritoneal [OFTPP] cancer). CP, common parameter; IP, independent parameters; MCIP, mixed common and independent parameters; MRIP, mixed random and independent parameters; OFTPP, ovarian, fallopian tube, and primary peritoneal; OS, overall survival; PFS, progression-free survival; RCC, renal cell carcinoma; RP, random parameters.

Figure 4 depicts the results obtained from performing multi-indication meta-analyses using the data that were available on PFS and OS at the time of 2 NICE TAs: TA178 for RCC (Figure 4a) and TA285 for OFTPP cancer (Figure 4b). Further results, including numerical estimates, are presented in Appendix E.2.1 and Appendix E.3.1 in Supplemental material, respectively. Broadly, the point estimates obtained from the different models are consistent with one another within the target indication, and there is large overlap in the CrIs for the effects on both PFS and OS. The estimates from the sharing models (CP, MCIP, RP, MRIP) show improved precision compared with the IP model, which does not allow for sharing across indications.

The results from the TA time point analyses show a similar trend to those obtained from the analysis of present-day data, in terms of the benefit of sharing across indications for gaining precision in effect estimates. However, less evidence had accumulated at these earlier time points, and so the corresponding analyses provide less precise predicted OS effect estimates (using both the univariate and bivariate approaches). In particular, the predicted OS effect estimates obtained from the bivariate models are noticeably more imprecise compared with the pooled estimates from the univariate models. This finding remains even when sharing is imposed on both the PFS effect and the surrogate relationship. This contrast with the results from the present-day analysis suggests that predictions based on the bivariate models are unlikely to strengthen estimates in realistic appraisal contexts.

## Discussion

In this article, we explored a range of meta-analysis models for the synthesis of evidence on treatment effects on an individual endpoint or on a surrogate relationship between treatment effects on a surrogate endpoint and a final clinical outcome, across multiple indications in oncology. Some of these models offer flexibility in terms of regulating the degree of sharing of information across indications, depending on the degree of similarity of the indication-level model parameters, which may be particularly useful in the HTA decision-making process. Our work represents the first application of the models in this context. We demonstrated the methods using data on treatment effects of bevacizumab on PFS and OS from trials across multiple cancer indications, by applying them to all data available at present day and, in the context of HTA, to data that had accumulated at time points corresponding to 2 NICE TAs of bevacizumab, for renal and ovarian cancers. Our case study showed that these methods can significantly strengthen inferences and therefore have an important role in support of policy (i.e., in terms of implications for NICE recommendations and price negotiations).

Bevacizumab is one of the first examples of a targeted multi-indication cancer drug, and therefore, its evidence base is relatively well developed at present day. Such evidence still shows considerable uncertainty in the OS effect estimates within most of the indications for which bevacizumab is licensed but does not show significant heterogeneity in effects across indications (see Figure 1). This suggests that a case study on bevacizumab is appropriate to explore whether sharing information across indications has the potential to strengthen indication-specific inference in the HTA context.

Our case study showed that the application of methods for sharing information across indications can lead to more precise inferences on treatment effects and on the parameters describing a surrogate relationship. This gain in precision was more noticeable for indications with limited evidence. Although the OS effects predicted using the surrogacy parameters were consistent with the pooled OS effects (from sharing across indications on OS effects alone), they showed meaningful gains in precision only when sharing was imposed on the PFS effects in addition to the surrogacy parameters. Corresponding analyses using the earlier, sparser (within and across indications) evidence available at particular HTA appraisal points showed that sharing on surrogacy parameters, even when also sharing on PFS effects, did not notably reduce uncertainty in predicted OS effects. These findings highlight that uncertainty in the surrogacy parameter estimates may be due to limited evidence or the lack of a clear surrogate relationship within the broader evidence base (i.e., within other indications), and this must be considered when determining whether sharing across indications on these parameters is appropriate.

There were differences in the support provided by the indication-level evidence for surrogacy between indications with higher levels of evidence. For example, the evidence suggested a surrogate relationship within CRC (9 trials), with small, precise estimates for the intercept and conditional variance parameters and a precise nonzero estimate for the slope parameter. Such estimates are indicative of surrogacy, although there are no clear-cut rules to infer this statistically. Despite there being a similar level of evidence in BC (10 trials), there was higher uncertainty in the slope and conditional variance parameters, which implies that surrogacy may not hold within this indication. Thus, the surrogacy parameter estimates for BC based on sharing information from other indications such as CRC may be deemed overly precise. However, it can be argued that as long as these results are not used to judge the strength of the surrogate relationship within BC alone, this sharing can be implemented to improve our predictions of the effect on the final outcome in other indications. We performed a sensitivity analysis by applying the sharing models to the present-day data after removing trials in BC but did not see a notable difference in the surrogacy parameter estimates in the remaining indications (see Appendix E.7.1 in Supplemental material).

The predicted OS effect estimate was more greatly influenced by the precision of the corresponding PFS effect estimate, which was entered into the bivariate model to make the prediction, than the surrogacy parameter estimates themselves. This suggests that applying a bivariate model to predict an OS effect may be applicable only where there is strong evidence on the PFS effect. In an HTA context, the feasibility of predicting the OS effect for a particular indication will depend on the level of evidence available on PFS within that indication and across other indications from which sharing is deemed plausible. Further research is required to explore the conditions under which sharing on surrogate relationships strengthens the predictions of OS effects. We expect that this is likely to require that at least 1 indication in the broader, multi-indication evidence base has a meaningful level of evidence (in terms of data on treatment effects on both the surrogate endpoint and the final outcome) and shows support for surrogacy.

In this work, we explored a range of models imposing different levels of information sharing, including no sharing across indications and models with increasing levels of flexibility in the strength of sharing, to assuming an equal, common, effect across all indications. These models fit the data similarly when sharing on each endpoint individually (univariate models), adding to the plausibility of sharing across all indications and suggesting that the added flexibility of the models with higher parametrizations was not required. However, the application of the more flexible sharing (mixture) models requires important methodological considerations when sharing on surrogate relationships (bivariate models). For instance, the mixture probabilities were much lower for the conditional variance parameter compared with the intercept and slope parameters, cautioning against sharing on this parameter. This may also explain why the mixture models provided a better fit to these data compared with the nonmixture models, which make more rigid assumptions regarding sharing. We performed a sensitivity analysis where the sharing models were implemented with the assumption of independent conditional variance parameters (i.e., sharing on the intercept and slope parameters only) but did not see a notable difference in results (see Appendix E.6.1 in Supplemental material).

A better understanding of the scenarios in which the mixture models offer a benefit over the nonmixture models, for example, where data from a particular indication are more extreme relative to the other indications, would be provided by a simulation study. Some of such scenarios for sharing information on surrogate relationships across treatment classes (strata) have been explored by Papanikos et al.^15^ in a simulation study. They found, for example, that where the slope of the surrogate relationship was different in one treatment class compared with others, the full exchangeability model resulted in a more biased slope compared with the mixture (partial exchangeability) model and that probabilities of exchageability were markedly lower for this particular class. They also found that at least 3 strata (i.e., treatment classes or indications) are required to apply the partial exchangeability (mixture) model and report that the factors affecting the number of studies, and the number of strata, required to apply the models include the magnitude of between-trials heterogeneity, the magnitude of between-strata heterogeneity, and similarity in the surrogate relationships across strata. In the multi-indication meta-analysis context, data requirements (in terms of the numbers of indications and trials in the synthesis) will critically depend on the parameter being summarized. For example, applying models to share information on effects on a single endpoint (e.g., OS) is likely to require less data than sharing on the surrogate relationship between endpoints. Furthermore, more complex models, such as the mixture models, include a greater number of parameters and therefore will require more data to estimate these precisely. The presence of large between-trial heterogeneity in the evidence will also indicate that more data are needed to apply the models at the same level of sharing.

To illustrate and explore the sharing methods in a multi-indication evidence synthesis context, in our case study, we considered it appropriate to focus the synthesis on aggregate trial-level data on a treatment effect for a survival outcome, represented by a log HR on either PFS or OS. We extracted these effect estimates from the literature as reported in the trial publications, where they are typically obtained from a time-to-event analysis assuming proportional hazards. However, future research on multi-indication meta-analyses should extend the methodological framework to account for scenarios in which the log HR is not representative of the true effect, for example, where there is evidence of nonproportional hazards between trial arms. Access to individual participant data would help to relax the proportional hazards assumption and unify the methodology used across trials.

In this case study on bevacizumab, the evidence did not suggest the presence of significant heterogeneity in the effect estimates across trials. A high level of heterogeneity in the evidence base can be due to a number of clinical and methodological factors. For example, we assumed an additive treatment effect for bevacizumab, but cases in which the effect of the treatment is synergistic (due to interaction with background therapy) can introduce heterogeneity. The data did not suggest effect modification from background therapy, and the plausibility of an additive effect of bevacizumab was confirmed by a clinical expert. A number of trials in our case study included crossover and were completed before the publication of NICE guidance on methods to adjust for treatment switching,^26^ which may have led to differences in the adjustments made to individual trial estimates. Similarly, the definition of disease progression differed across some trials (e.g., based on an independent review facility v. investigator assessment), which can introduce heterogeneity in the effect estimates on PFS, and this was not accounted for in our analysis. We did not take into consideration differences in the follow-up period across trials and indications. Furthermore, there can be variability in the trial populations as trials recruit participants from different subgroups within a particular indication (e.g., HER2 status in breast cancer). In other case studies, these factors may lead to significant heterogeneity, which will need to be accounted for in the meta-analysis.

In our analysis, we implemented methods for sharing information on the surrogate relationship between treatment effects on PFS and OS across indications. The biological plausibility of sharing across indications (or populations) may depend on molecular or genomic factors, including predictive biomarkers. However, such biomarkers are not always measured consistently across populations. To determine whether it is appropriate to share information on the strength of a surrogate relationship across indications, consideration should be given to the population characteristics. In particular, an assessment of which characteristics are likely to affect treatment effectiveness, whether there are important differences in these characteristics across indications, and whether their impact on the effectiveness is proportional on both the surrogate endpoint and the final clinical outcome. Ultimately, any analyses supporting policy should be grounded in the biological plausibility of the surrogate relationship^27,28^ and, in this case, for both within indications and the similarity of surrogacy patterns across indications, the minimum level of evidence in surrogate endpoint evaluations.

The findings from this case study on bevacizumab are likely to be applicable to other multi-indication health technologies. In our case study, we focused on synthesizing evidence across its licensed indications, aiming to demonstrate multi-indication meta-analysis methods in an appraisal context where the interest is in predicting an effect in a target indication for which a drug has been licensed. Evidence from unlicensed indications is likely to show reduced relative effectiveness compared with licensed indications; thus, its inclusion in univariate meta-analysis would hinder the assumption of exchangeability of the treatment effects across the 2 sets of indications. The inclusion of data from unlicensed indications could improve the evidence base for establishing a surrogate relationship, where the exchangeability of surrogacy parameters, rather than treatment effects, is assumed across indications. However, the plausibility of the different sharing assumptions should be carefully explored with clinical experts, where possible, particularly as the quantity of data available in practice is unlikely to be sufficient to examine this. Further research should explore how this information can be reasonably elicited and combined with data-driven approaches based on model fit. For example, leave-one-out cross-validation can be used to compare different models in terms of the predictions of the effect on the final outcome. When assessing more complex models, good predictions can be an important justification for model choice, supporting clinical experts in expressing evidence-based opinions.

## Supplemental Material

sj-pdf-1-mdm-10.1177_0272989X241295665 – Supplemental material for Multi-indication Evidence Synthesis in Oncology Health Technology Assessment: Meta-analysis Methods and Their Application to a Case Study of Bevacizumab Supplemental material, sj-pdf-1-mdm-10.1177_0272989X241295665 for Multi-indication Evidence Synthesis in Oncology Health Technology Assessment: Meta-analysis Methods and Their Application to a Case Study of Bevacizumab by Janharpreet Singh, Sumayya Anwer, Stephen Palmer, Pedro Saramago, Anne Thomas, Sofia Dias, Marta O Soares and Sylwia Bujkiewicz in Medical Decision Making
