# Supplementary material for: Multi-indication Evidence Synthesis in Oncology Health Technology Assessment: Meta-analysis Methods and Their Application to a Case Study of Bevacizumab
Source: Med Decis Making. 2024 Nov 18;45(1):17–33. doi: 10.1177/0272989X241295665 (PMC11645851; doi:10.1177/0272989X241295665)
Supplement: sj-pdf-1-mdm-10.1177_0272989X241295665 – Supplemental material for Multi-indication Evidence Synthesis in Oncology Health Technology Assessment: Meta-analysis Methods and Their Application to a Case Study of Bevacizumab [file sj-pdf-1-mdm-10.1177_0272989X241295665.pdf]

A Case study: bevacizumab

Figure A1 shows the development of RCT evidence on the relative effectiveness of bevacizumab from 1997 to 2023, and the time point(s) at which these trials reported results. Evidence sources included NICE TAs, Cochrane Reviews, and the *ClinicalTrials.gov* database. The scope was restricted to phase II/III trials where the treatment effect of bevacizumab as a monotherapy could be isolated. As such, we only considered trials in which the sole difference between the treatment allocated to the intervention and control arms was the addition of bevacizumab to background therapy. These effect estimates were extracted from trial publications reporting the results of the main analysis, which included stratification or regression estimates, and/or adjustment for participant cross-over. Where there were effect estimates reported at multiple time points for a particular trial, the latest reported estimate was selected for our analysis.

Over the time period considered, bevacizumab was the subject of seven NICE TAs across four cancer indications. The appraisal time points are indicated in Figure A1 with vertical dashed lines. These appraisal time points are based on the publication date of the NICE final appraisal document corresponding to each TA. For our analyses, we will focus on the evidence available at present day and at two appraisal time points, an earlier time point with limited evidence and a later time point with more abundant evidence. The first appraisal is TA178, appraising the use of bevacizumab in RCC [27]. At the time of this appraisal, there were three completed trials in the target indication (three reporting effect estimates for PFS and two for OS), and an additional 14 trials across three other indications (14 reporting effect estimates for PFS and 11 for OS). This was the first appraisal on bevacizumab to include data from at least three indications, thus allowing the application of the hierarchical multi-indication meta-analysis models. The second appraisal is TA285 in ovarian cancer [28]. By the time of this appraisal, there were four trials in this indication (all reporting effect estimates for both PFS and OS), and an additional 28 trials across six other indications (28 reporting effect estimates for PFS, and 23 for OS). We focus on these time points to demonstrate the methods in contrasting scenarios in terms of the availability of evidence.

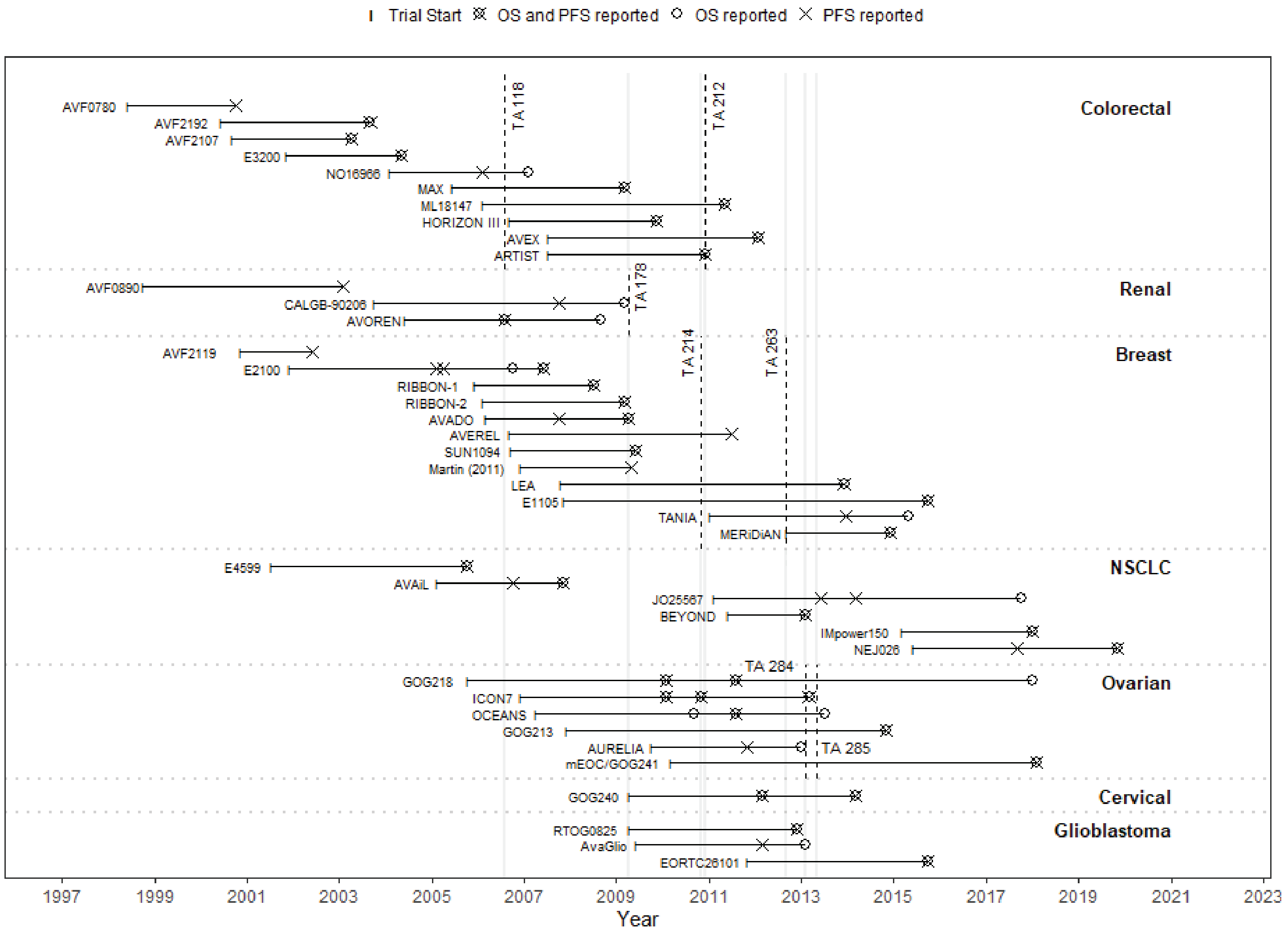

Figure A1: Plot summarises the accumulation of data on overall survival (OS) (circles) and progression-free survival (PFS) (crosses) from randomised controlled trials assessing bevacizumab within each cancer indication (labelled on far right) between 1997 and 2023. Vertical dashed lines indicate time points corresponding to particular technology appraisals (TAs) by the National Institute for Health and Care Excellence. NSCLC - Non-small cell lung cancer.

B Abbreviations

B.1 Treatment names

Table B1: List of abbreviations for treatments listed in Figure 1.

Treatment name	Abbreviation
Paclitaxel	PAC
Bevacizumab	BEV
Docetaxel	DOC
Capecitabine	CAP
Taxane	TAX
Anthracycline	ANT
Cisplatin	CIS
Topotecan	TOP
Irinotecan	IRI
Fluorouracil	FLU
Leucovorin	LEU
Oxaliplatin	OXA
Lomustine	LOM
Temozolomide	TEM
Carboplatin	CAR
Gemcitabine	GEM
Erlotinib	ERL
Atezolizumab	ATE
Interferon	INT
Chemotherapy	Chemo
Placebo	PBO
Radiotherapy	Rad
Trastuzumab	TRA
Endocrine Therapy	ET
Sunitinib	SUN
Cediranib	CED

C Formulae for predicting estimates of a treatment effect on overall survival

A predicted treatment effect estimate on overall survival (OS) can be obtained by entering the indication-specific progression-free survival (PFS) effect estimate into the model describing the surrogate relationship for a particular indication j. To account for uncertainty in the PFS estimate, the posterior mean $\hat{d}_{j,PFS}$ and posterior standard deviation $\hat{\sigma}^2_{j,PFS}$ are used to obtain a sampled value,

$$d_{j,PFS} \sim N(\hat{d}_{j,PFS}, \hat{\sigma}^2_{d_{j,PFS}}), \tag{12}$$

where $d_{j,PFS}$ represents the sampled PFS estimate.

The sampled PFS estimate is entered into the regression equation describing the surrogate relationship (i.e. intercept and slope) in indication j,

$$d_{j,OS} = \lambda_{0j} + \lambda_{1j} d_{j,PFS} \tag{13}$$

where $d_{j,OS}$ is the predicted treatment effect on OS.

D Appraisal time point data sets

D.1 NICE TA178

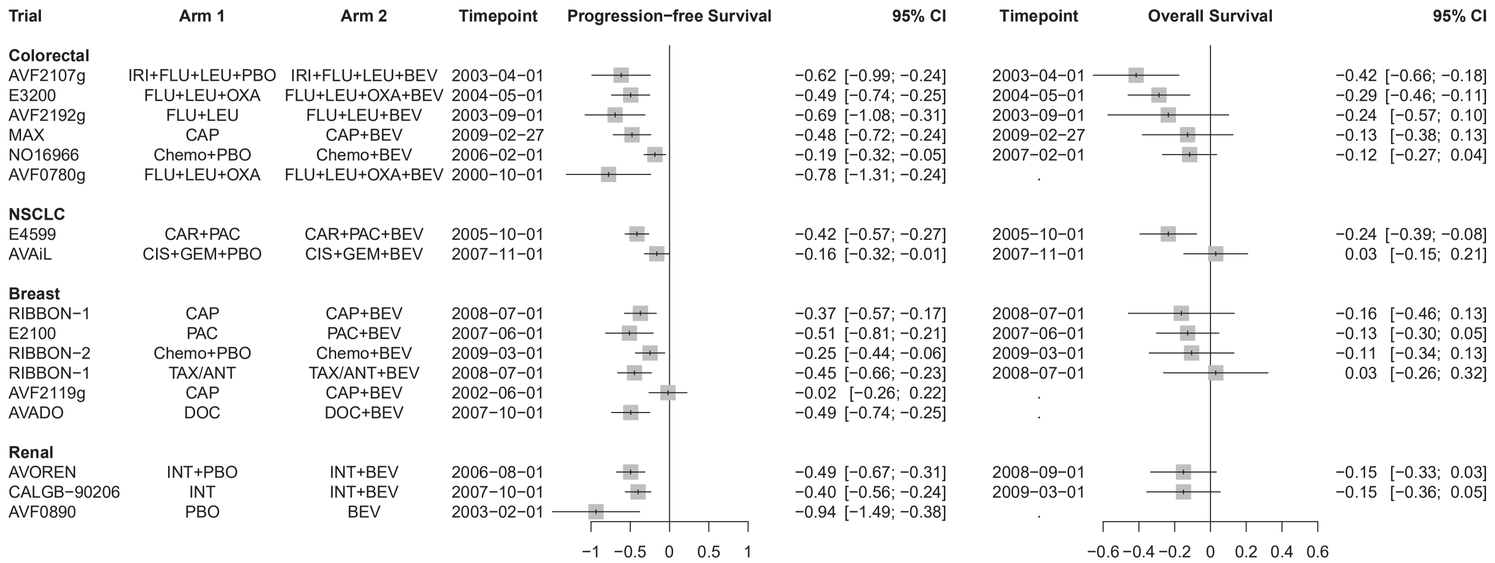

Figure D1: Forest plot summarising log-transformed hazard ratio estimates for effects on progression-free survival and overall survival from randomised controlled trials assessing bevacizumab across cancer indications, for the data available at the time of NICE TA178. Estimates are ordered by OS effect size within each cancer indication. BEV - bevacizumab. NSCLC - Non-small cell lung cancer. OFTPP - Ovarian, fallopian tube and primary peritoneal.

D.2 NICE TA285

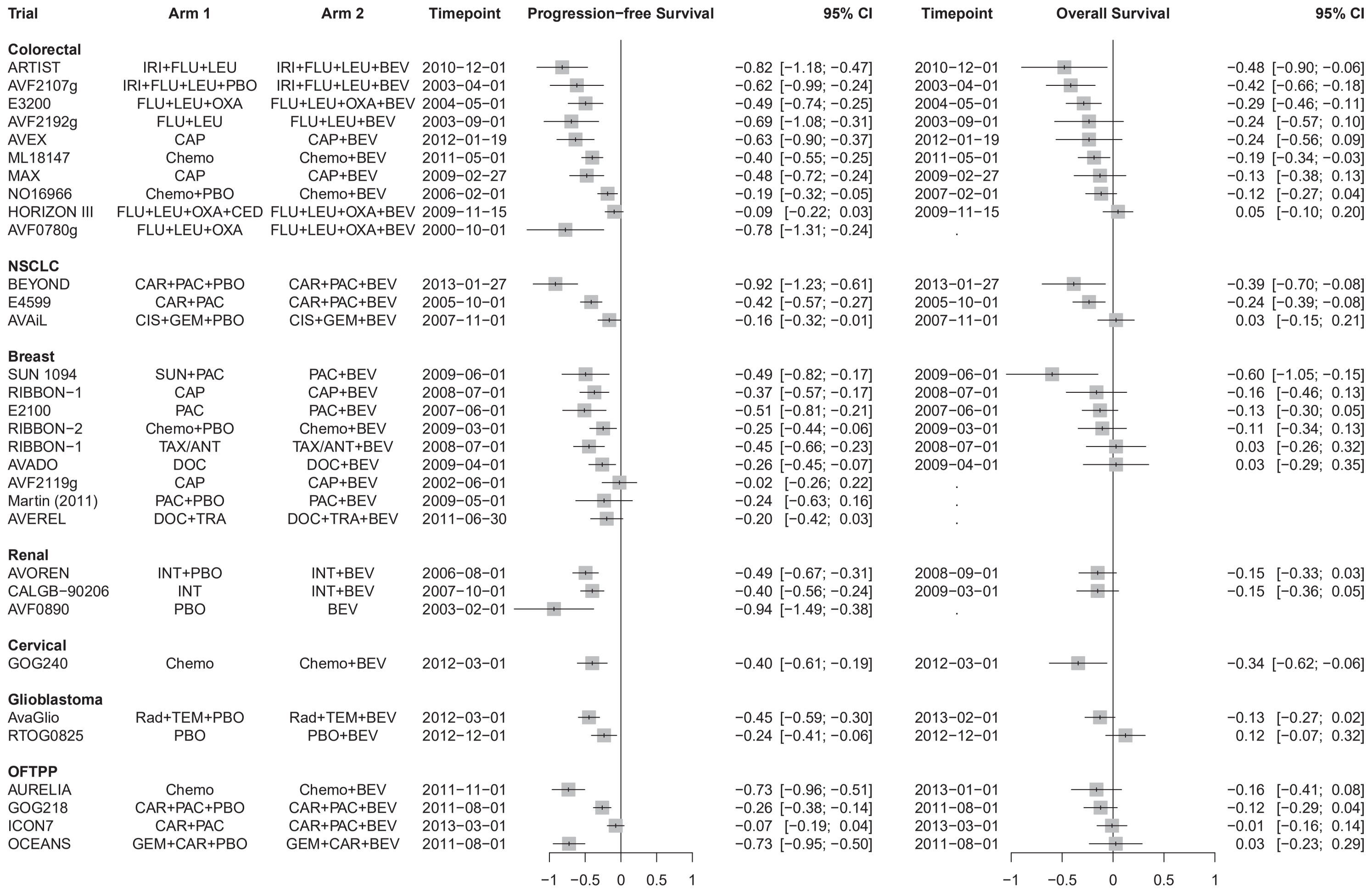

Trial	Arm 1	Arm 2	Timepoint	Progression−free Survival 95% CI	Timepoint	Overall Survival 95% CI
**Colorectal**						
ARTIST	IRI+FLU+LEU	IRI+FLU+LEU+BEV	2010−12−01	−0.82 [−1.18; −0.47]	2010−12−01	−0.48 [−0.90; −0.06]
AVF2107g	IRI+FLU+LEU+PBO	IRI+FLU+LEU+BEV	2003−04−01	−0.62 [−0.99; −0.24]	2003−04−01	−0.42 [−0.66; −0.18]
E3200	FLU+LEU+OXA	FLU+LEU+OXA+BEV	2004−05−01	−0.49 [−0.74; −0.25]	2004−05−01	−0.29 [−0.46; −0.11]
AVF2192g	FLU+LEU	FLU+LEU+BEV	2003−09−01	−0.69 [−1.08; −0.31]	2003−09−01	−0.24 [−0.57; 0.10]
AVEX	CAP	CAP+BEV	2012−01−19	−0.63 [−0.90; −0.37]	2012−01−19	−0.24 [−0.56; 0.09]
ML18147	Chemo	Chemo+BEV	2011−05−01	−0.40 [−0.55; −0.25]	2011−05−01	−0.19 [−0.34; −0.03]
MAX	CAP	CAP+BEV	2009−02−27	−0.48 [−0.72; −0.24]	2009−02−27	−0.13 [−0.38; 0.13]
NO16966	Chemo+PBO	Chemo+BEV	2006−02−01	−0.19 [−0.32; −0.05]	2007−02−01	−0.12 [−0.27; 0.04]
HORIZON III	FLU+LEU+OXA+CED	FLU+LEU+OXA+BEV	2009−11−15	−0.09 [−0.22; 0.03]	2009−11−15	0.05 [−0.10; 0.20]
AVF0780g	FLU+LEU+OXA	FLU+LEU+OXA+BEV	2000−10−01	−0.78 [−1.31; −0.24]	.	
**NSCLC**						
BEYOND	CAR+PAC+PBO	CAR+PAC+BEV	2013−01−27	−0.92 [−1.23; −0.61]	2013−01−27	−0.39 [−0.70; −0.08]
E4599	CAR+PAC	CAR+PAC+BEV	2005−10−01	−0.42 [−0.57; −0.27]	2005−10−01	−0.24 [−0.39; −0.08]
AVAiL	CIS+GEM+PBO	CIS+GEM+BEV	2007−11−01	−0.16 [−0.32; −0.01]	2007−11−01	0.03 [−0.15; 0.21]
**Breast**						
SUN 1094	SUN+PAC	PAC+BEV	2009−06−01	−0.49 [−0.82; −0.17]	2009−06−01	−0.60 [−1.05; −0.15]
RIBBON−1	CAP	CAP+BEV	2008−07−01	−0.37 [−0.57; −0.17]	2008−07−01	−0.16 [−0.46; 0.13]
E2100	PAC	PAC+BEV	2007−06−01	−0.51 [−0.81; −0.21]	2007−06−01	−0.13 [−0.30; 0.05]
RIBBON−2	Chemo+PBO	Chemo+BEV	2009−03−01	−0.25 [−0.44; −0.06]	2009−03−01	−0.11 [−0.34; 0.13]
RIBBON−1	TAX/ANT	TAX/ANT+BEV	2008−07−01	−0.45 [−0.66; −0.23]	2008−07−01	0.03 [−0.26; 0.32]
AVADO	DOC	DOC+BEV	2009−04−01	−0.26 [−0.45; −0.07]	2009−04−01	0.03 [−0.29; 0.35]
AVF2119g	CAP	CAP+BEV	2002−06−01	−0.02 [−0.26; 0.22]	.	
Martin (2011)	PAC+PBO	PAC+BEV	2009−05−01	−0.24 [−0.63; 0.16]	.	
AVEREL	DOC+TRA	DOC+TRA+BEV	2011−06−30	−0.20 [−0.42; 0.03]	.	
**Renal**						
AVOREN	INT+PBO	INT+BEV	2006−08−01	−0.49 [−0.67; −0.31]	2008−09−01	−0.15 [−0.33; 0.03]
CALGB−90206	INT	INT+BEV	2007−10−01	−0.40 [−0.56; −0.24]	2009−03−01	−0.15 [−0.36; 0.05]
AVF0890	PBO	BEV	2003−02−01	−0.94 [−1.49; −0.38]	.	
**Cervical**						
GOG240	Chemo	Chemo+BEV	2012−03−01	−0.40 [−0.61; −0.19]	2012−03−01	−0.34 [−0.62; −0.06]
**Glioblastoma**						
AvaGlio	Rad+TEM+PBO	Rad+TEM+BEV	2012−03−01	−0.45 [−0.59; −0.30]	2013−02−01	−0.13 [−0.27; 0.02]
RTOG0825	PBO	PBO+BEV	2012−12−01	−0.24 [−0.41; −0.06]	2012−12−01	0.12 [−0.07; 0.32]
**OFTPP**						
AURELIA	Chemo	Chemo+BEV	2011−11−01	−0.73 [−0.96; −0.51]	2013−01−01	−0.16 [−0.41; 0.08]
GOG218	CAR+PAC+PBO	CAR+PAC+BEV	2011−08−01	−0.26 [−0.38; −0.14]	2011−08−01	−0.12 [−0.29; 0.04]
ICON7	CAR+PAC	CAR+PAC+BEV	2013−03−01	−0.07 [−0.19; 0.04]	2013−03−01	−0.01 [−0.16; 0.14]
OCEANS	GEM+CAR+PBO	GEM+CAR+BEV	2011−08−01	−0.73 [−0.95; −0.50]	2011−08−01	0.03 [−0.23; 0.29]

Figure D2: Forest plot summarising log-transformed hazard ratio estimates for effects on progression-free survival and overall survival from randomised controlled trials assessing bevacizumab across cancer indications, for the data available at the time of NICE TA285. Estimates are ordered by OS effect size within each cancer indication. BEV - bevacizumab. NSCLC - Non-small cell lung cancer. OFTPP - Ovarian, fallopian tube and primary peritoneal.

E Additional results

E.1 Present day

E.1.1 Pooled OS and PFS estimates

Table E1: Median and 95% credible interval estimates for overall survival using data available at present-day. IP - Independent parameters, CP - Common parameters, MCIP - Mixed common and independent parameters, RP - Random parameters, MRIP - Mixed random and independent parameters, BC - Breast cancer, CC - Cervical cancer, CRC - Colorectal cancer, GBM - Glioblastoma, NSCLC - Non-small cell lung cancer, OFTPP - Ovarian, fallopian tube, and primary peritoneal, RCC - Renal cell carcinoma.

Indication	IP	CP	MCIP	RP	MRIP
CRC	-0.19 (-0.33, -0.07)	-0.12 (-0.17, -0.07)	-0.12 (-0.18, -0.06)	-0.14 (-0.25, -0.07)	-0.14 (-0.25, -0.06)
RCC	-0.15 (-0.65, 0.35)	-0.12 (-0.17, -0.07)	-0.12 (-0.18, -0.06)	-0.12 (-0.25, -0.01)	-0.13 (-0.25, -0.01)
BC	-0.11 (-0.22, 0.00)	-0.12 (-0.17, -0.07)	-0.12 (-0.17, -0.06)	-0.12 (-0.19, -0.04)	-0.12 (-0.19, -0.04)
NSCLC	-0.14 (-0.34, 0.08)	-0.12 (-0.17, -0.07)	-0.12 (-0.18, -0.06)	-0.12 (-0.23, -0.02)	-0.12 (-0.23, -0.02)
OFTPP	-0.07 (-0.19, 0.04)	-0.12 (-0.17, -0.07)	-0.12 (-0.17, -0.06)	-0.10 (-0.18, -0.01)	-0.10 (-0.18, -0.01)
CC	-0.26 (-1.35, 0.83)	-0.12 (-0.17, -0.07)	-0.12 (-0.18, -0.06)	-0.13 (-0.30, 0.01)	-0.13 (-0.31, 0.01)
GBM	-0.03 (-0.36, 0.32)	-0.12 (-0.17, -0.07)	-0.12 (-0.18, -0.06)	-0.10 (-0.21, 0.03)	-0.10 (-0.21, 0.04)
All Indications		-0.12 (-0.17, -0.07)	-0.12 (-0.17, -0.06)	-0.12 (-0.20, -0.04)	-0.12 (-0.20, -0.04)
New Indication				-0.12 (-0.29, 0.05)	-0.12 (-0.29, 0.04)

Table E2: Median and 95% credible interval estimates for progression-free survival using data available at present-day. IP - Independent parameters, CP - Common parameters, MCIP - Mixed common and independent parameters, RP - Random parameters, MRIP - Mixed random and independent parameters, BC - Breast cancer, CC - Cervical cancer, CRC - Colorectal cancer, GBM - Glioblastoma, NSCLC - Non-small cell lung cancer, OFTPP - Ovarian, fallopian tube, and primary peritoneal, RCC - Renal cell carcinoma.

Indication	IP	CP	MCIP	RP	MRIP
CRC	-0.47 (-0.67, -0.30)	-0.37 (-0.45, -0.30)	-0.37 (-0.47, -0.30)	-0.42 (-0.58, -0.30)	-0.42 (-0.58, -0.31)
RCC	-0.50 (-1.01, -0.15)	-0.37 (-0.45, -0.30)	-0.37 (-0.47, -0.30)	-0.42 (-0.61, -0.28)	-0.42 (-0.62, -0.28)
BC	-0.29 (-0.38, -0.21)	-0.37 (-0.45, -0.30)	-0.37 (-0.45, -0.29)	-0.32 (-0.42, -0.24)	-0.32 (-0.42, -0.24)
NSCLC	-0.41 (-0.73, -0.11)	-0.37 (-0.45, -0.30)	-0.37 (-0.46, -0.30)	-0.40 (-0.58, -0.25)	-0.40 (-0.57, -0.25)
OFTPP	-0.42 (-0.71, -0.11)	-0.37 (-0.45, -0.30)	-0.37 (-0.46, -0.30)	-0.40 (-0.57, -0.25)	-0.40 (-0.57, -0.25)
CC	-0.38 (-1.48, 0.70)	-0.37 (-0.45, -0.30)	-0.37 (-0.47, -0.30)	-0.39 (-0.61, -0.20)	-0.39 (-0.61, -0.20)
GBM	-0.46 (-0.94, 0.01)	-0.37 (-0.45, -0.30)	-0.37 (-0.47, -0.30)	-0.41 (-0.60, -0.25)	-0.41 (-0.60, -0.25)
All Indications		-0.37 (-0.45, -0.30)	-0.37 (-0.46, -0.30)	-0.40 (-0.53, -0.29)	-0.39 (-0.53, -0.29)
New Indication				-0.39 (-0.67, -0.15)	-0.39 (-0.66, -0.16)

E.1.2 Predicted OS estimates

Table E3: Median and 95% credible interval estimates for predicted overall survival, from entering the independent parameters (IP) progression-free survival estimate into the surrogacy models, using data available at present-day. IP - Independent parameters, CP - Common parameters, MCIP - Mixed common and independent parameters, RP - Random parameters, MRIP - Mixed random and independent parameters, BC - Breast cancer, CC - Cervical cancer, CRC - Colorectal cancer, GBM - Glioblastoma, NSCLC - Non-small cell lung cancer, OFTPP - Ovarian, fallopian tube, and primary peritoneal, RCC - Renal cell carcinoma.

Indication	IP	CP	MCIP	RP	MRIP
CRC	-0.22 (-0.36, -0.09)	-0.17 (-0.25, -0.07)	-0.17 (-0.26, -0.08)	-0.20 (-0.31, -0.09)	-0.20 (-0.31, -0.09)
RCC	-0.16 (-9.76, 10.42)	-0.13 (-5.06, 4.07)	-0.14 (-4.86, 3.96)	-0.15 (-4.55, 4.22)	-0.15 (-4.87, 4.57)
BC	-0.09 (-0.23, 0.05)	-0.12 (-0.26, 0.00)	-0.12 (-0.26, 0.00)	-0.10 (-0.24, 0.03)	-0.10 (-0.24, 0.03)
NSCLC	-0.14 (-0.38, 0.11)	-0.12 (-0.26, 0.06)	-0.12 (-0.27, 0.06)	-0.13 (-0.30, 0.06)	-0.13 (-0.31, 0.06)
OFTPP	-0.08 (-0.23, 0.09)	-0.07 (-0.19, 0.06)	-0.07 (-0.19, 0.06)	-0.08 (-0.20, 0.06)	-0.08 (-0.20, 0.06)
CC	-0.25 (-33.56, 32.95)	-0.23 (-7.74, 7.33)	-0.23 (-8.22, 7.80)	-0.25 (-9.94, 9.24)	-0.25 (-10.45, 9.71)
GBM	-0.03 (-0.94, 0.91)	0.00 (-0.62, 0.60)	0.00 (-0.60, 0.59)	-0.01 (-0.63, 0.61)	-0.01 (-0.63, 0.62)

Table E4: Median and 95% credible interval estimates for predicted overall survival, from entering the model-specific progression-free survival estimate into the corresponding surrogacy models, using data available at present-day. IP - Independent parameters, CP - Common parameters, MCIP - Mixed common and independent parameters, RP - Random parameters, MRIP - Mixed random and independent parameters, BC - Breast cancer, CC - Cervical cancer, CRC - Colorectal cancer, GBM - Glioblastoma, NSCLC - Non-small cell lung cancer, OFTPP - Ovarian, fallopian tube, and primary peritoneal, RCC - Renal cell carcinoma.

Indication	IP	CP	MCIP	RP	MRIP
CRC	-0.22 (-0.36, -0.09)	-0.11 (-0.16, -0.06)	-0.12 (-0.19, -0.07)	-0.19 (-0.29, -0.10)	-0.19 (-0.29, -0.10)
RCC	-0.16 (-9.76, 10.42)	-0.11 (-0.16, -0.06)	-0.12 (-0.18, -0.06)	-0.14 (-0.27, -0.02)	-0.14 (-0.28, -0.01)
BC	-0.09 (-0.23, 0.05)	-0.11 (-0.16, -0.06)	-0.12 (-0.17, -0.06)	-0.09 (-0.17, -0.01)	-0.09 (-0.18, -0.01)
NSCLC	-0.14 (-0.38, 0.11)	-0.11 (-0.16, -0.06)	-0.11 (-0.17, -0.06)	-0.14 (-0.25, -0.04)	-0.14 (-0.26, -0.03)
OFTPP	-0.08 (-0.23, 0.09)	-0.11 (-0.16, -0.06)	-0.12 (-0.17, -0.05)	-0.08 (-0.18, 0.00)	-0.08 (-0.18, 0.01)
CC	-0.25 (-33.56, 32.95)	-0.11 (-0.16, -0.06)	-0.12 (-0.24, -0.04)	-0.16 (-0.37, -0.01)	-0.15 (-0.40, 0.03)
GBM	-0.03 (-0.94, 0.91)	-0.11 (-0.16, -0.06)	-0.12 (-0.17, -0.04)	-0.07 (-0.19, 0.05)	-0.07 (-0.21, 0.06)
All Indications		-0.11 (-0.16, -0.06)	-0.12 (-0.17, -0.06)	-0.13 (-0.23, -0.03)	-0.12 (-0.23, -0.03)
New Indication				-0.12 (-0.38, 0.09)	-0.12 (-0.38, 0.10)

E.1.3 Deviance information criterion (DIC)

Table E5: Residual deviance, deviance, number of effective parameters, and deviance information criterian (DIC) from application of models to data on overall survival at present-day. *Based on 38 data points. IP - Independent parameters, CP - Common parameter, MCIP - Mixed common and independent parameters, RP - Random parameters, MRIP - Mixed random and independent parameters

Model	Residual deviance	Deviance	p_D	DIC
IP	33.58	-39.04	20.26	-18.78
CP	33.80	-38.82	17.06	-21.76
MCIP	33.79	-38.83	17.10	-21.73
RP	33.38	-39.24	17.87	-21.37
MRIP	33.42	-37.81	17.89	-19.92

Table E6: Residual deviance, deviance, number of effective parameters, and deviance information criterian (DIC) from application of models to data on progression-free survival at present-day. *Based on 43 data points. IP - Independent parameters, CP - Common parameter, MCIP - Mixed common and independent parameters, RP - Random parameters, MRIP - Mixed random and independent parameters

Model	Residual deviance	Deviance	p_D	DIC
IP	42.48	-44.76	29.17	-15.59
CP	45.28	-41.97	28.96	-13.00
MCIP	45.19	-42.05	28.93	-13.13
RP	43.44	-43.80	28.35	-15.45
MRIP	43.48	-42.38	28.28	-14.10

E.1.4 Surrogacy parameter estimates

Table E7: Median and 95% credible interval estimates for intercepts from surrogacy using data available at present-day. IP - Independent parameters, CP - Common parameters, MCIP - Mixed common and independent parameters, RP - Random parameters, MRIP - Mixed random and independent parameters, BC - Breast cancer, CC - Cervical cancer, CRC - Colorectal cancer, GBM - Glioblastoma, NSCLC - Non-small cell lung cancer, OFTPP - Ovarian, fallopian tube, and primary peritoneal, RCC - Renal cell carcinoma.

Indication	N	IP	CP	MCIP	RP	MRIP
CRC	9	0.05 (-0.11, 0.21)	0.04 (-0.04, 0.12)	0.04 (-0.04, 0.13)	0.04 (-0.06, 0.12)	0.03 (-0.07, 0.13)
RCC	2	0.05 (-12.75, 15.19)	0.04 (-0.04, 0.12)	0.04 (-0.04, 0.13)	0.05 (-0.07, 0.16)	0.05 (-0.08, 0.17)
BC	10	0.04 (-0.33, 0.45)	0.04 (-0.04, 0.12)	0.04 (-0.04, 0.13)	0.05 (-0.06, 0.14)	0.04 (-0.06, 0.14)
NSCLC	6	0.10 (-0.18, 0.38)	0.04 (-0.04, 0.12)	0.04 (-0.04, 0.13)	0.05 (-0.06, 0.15)	0.05 (-0.06, 0.15)
OFTPP	7	0.02 (-0.18, 0.28)	0.04 (-0.04, 0.12)	0.04 (-0.04, 0.13)	0.05 (-0.04, 0.15)	0.05 (-0.05, 0.15)
CC	1	-0.40 (-22.89, 23.86)	0.04 (-0.04, 0.12)	0.04 (-0.05, 0.14)	0.03 (-0.12, 0.14)	0.03 (-0.13, 0.16)
GBM	3	0.11 (-1.52, 1.64)	0.04 (-0.04, 0.12)	0.04 (-0.04, 0.13)	0.07 (-0.04, 0.21)	0.06 (-0.05, 0.21)
Overall	38		0.04 (-0.04, 0.12)	0.04 (-0.04, 0.13)	0.05 (-0.05, 0.14)	0.05 (-0.05, 0.14)

Table E8: Median and 95% credible interval estimates for slopes from surrogacy using data available at present-day. IP - Independent parameters, CP - Common parameters, MCIP - Mixed common and independent parameters, RP - Random parameters, MRIP - Mixed random and independent parameters, BC - Breast cancer, CC - Cervical cancer, CRC - Colorectal cancer, GBM - Glioblastoma, NSCLC - Non-small cell lung cancer, OFTPP - Ovarian, fallopian tube, and primary peritoneal, RCC - Renal cell carcinoma.

Indication	N	IP	CP	MCIP	RP	MRIP
CRC	9	0.59 (0.24, 0.91)	0.42 (0.23, 0.61)	0.43 (0.22, 0.66)	0.53 (0.29, 0.74)	0.52 (0.28, 0.74)
RCC	2	0.44 (-28.08, 34.37)	0.42 (0.23, 0.61)	0.42 (0.22, 0.63)	0.43 (0.14, 0.72)	0.43 (0.12, 0.73)
BC	10	0.40 (-0.55, 1.60)	0.42 (0.23, 0.61)	0.42 (0.22, 0.63)	0.42 (0.15, 0.70)	0.42 (0.15, 0.69)
NSCLC	6	0.58 (0.02, 1.12)	0.42 (0.23, 0.61)	0.42 (0.22, 0.63)	0.48 (0.23, 0.70)	0.47 (0.22, 0.71)
OFTPP	7	0.24 (-0.20, 0.75)	0.42 (0.23, 0.61)	0.41 (0.21, 0.63)	0.34 (0.10, 0.57)	0.34 (0.09, 0.58)
CC	1	-0.47 (-54.65, 59.38)	0.42 (0.23, 0.61)	0.42 (0.21, 0.75)	0.49 (0.16, 0.92)	0.47 (0.09, 0.98)
GBM	3	0.30 (-3.14, 3.47)	0.42 (0.23, 0.61)	0.42 (0.20, 0.63)	0.34 (0.03, 0.60)	0.35 (0.01, 0.63)
Overall	38		0.42 (0.23, 0.61)	0.42 (0.22, 0.63)	0.43 (0.20, 0.66)	0.43 (0.19, 0.66)

Table E9: Median and 95% credible interval estimates for conditonal variances from surrogacy using data available at present-day. IP - Independent parameters, CP - Common parameters, MCIP - Mixed common and independent parameters, RP - Random parameters, MRIP - Mixed random and independent parameters, BC - Breast cancer, CC - Cervical cancer, CRC - Colorectal cancer, GBM - Glioblastoma, NSCLC - Non-small cell lung cancer, OFTPP - Ovarian, fallopian tube, and primary peritoneal, RCC - Renal cell carcinoma.

Indication	N	IP	CP	MCIP	RP	MRIP
CRC	9	0.05 (0.00, 0.17)	0.05 (0.00, 0.11)	0.05 (0.00, 0.13)	0.04 (0.00, 0.11)	0.04 (0.00, 0.13)
RCC	2	0.26 (0.01, 1.02)	0.05 (0.00, 0.11)	0.05 (0.00, 0.26)	0.04 (0.00, 0.15)	0.05 (0.00, 0.33)
BC	10	0.07 (0.00, 0.26)	0.05 (0.00, 0.11)	0.04 (0.00, 0.14)	0.04 (0.00, 0.13)	0.04 (0.00, 0.16)
NSCLC	6	0.08 (0.00, 0.34)	0.05 (0.00, 0.11)	0.05 (0.00, 0.15)	0.04 (0.00, 0.14)	0.05 (0.00, 0.17)
OFTPP	7	0.06 (0.00, 0.30)	0.05 (0.00, 0.11)	0.05 (0.00, 0.19)	0.04 (0.00, 0.14)	0.04 (0.00, 0.18)
CC	1	0.34 (0.02, 1.12)	0.05 (0.00, 0.11)	0.07 (0.01, 0.75)	0.05 (0.00, 0.19)	0.08 (0.00, 0.74)
GBM	3	0.23 (0.02, 0.91)	0.05 (0.00, 0.11)	0.08 (0.01, 0.48)	0.05 (0.00, 0.18)	0.07 (0.00, 0.39)
Overall	38		0.05 (0.00, 0.11)	0.05 (0.00, 0.13)		

E.1.5 Deviance information criterion (DIC)

Table E10: Residual deviance, deviance, number of effective parameters, and deviance information criterian (DIC) from application of surrogacy models to data at present-day. *Based on 38 data points. IP - Independent parameters, CP - Common parameter, MCIP - Mixed common and independent parameters, RP - Random parameters, MRIP - Mixed random and independent parameters

Model	Residual deviance	Deviance	p_D	DIC
IP	70.95	-127.59	48.75	-78.84
CP	72.75	-118.81	39.52	-79.29
MCIP	72.35	-115.95	41.27	-74.68
RP	71.92	-122.36	42.80	-79.56
MRIP	71.61	-114.92	43.01	-71.91

E.1.6 Mixture probabilities

Table E11: Mixture probability estimates (as mean and standard deviation), and informative and vague mixture component estimates (as median and 95% credible interval), for intercepts from application of surrogacy models to data at present-day. MCIP - Mixed common and independent parameters, MRIP - Mixed random and independent parameters, BC - Breast cancer, CC - Cervical cancer, CRC - Colorectal cancer, GBM - Glioblastoma, NSCLC - Non-small cell lung cancer, OFTPP - Ovarian, fallopian tube, and primary peritoneal, RCC - Renal cell carcinoma.

Model	Indication	Probability	Informative	Vague
MCIP	BC	1.00 (0.04)	0.04 (-0.04, 0.13)	0.14 (-61.89, 61.86)
MCIP	CC	0.98 (0.14)	0.04 (-0.04, 0.13)	0.04 (-61.76, 61.83)
MCIP	CRC	0.99 (0.07)	0.04 (-0.04, 0.13)	-0.03 (-62.00, 61.82)
MCIP	GBM	0.99 (0.11)	0.04 (-0.04, 0.13)	0.13 (-61.82, 62.14)
MCIP	NSCLC	1.00 (0.04)	0.04 (-0.04, 0.13)	-0.08 (-61.91, 61.88)
MCIP	OFTPP	1.00 (0.05)	0.04 (-0.04, 0.13)	0.11 (-62.11, 62.06)
MCIP	RCC	1.00 (0.05)	0.04 (-0.04, 0.13)	0.11 (-62.02, 62.11)
MRIP	BC	1.00 (0.04)	0.04 (-0.06, 0.14)	0.04 (-61.87, 61.87)
MRIP	CC	0.99 (0.10)	0.03 (-0.12, 0.16)	-0.07 (-62.06, 61.74)
MRIP	CRC	1.00 (0.05)	0.03 (-0.07, 0.13)	0.02 (-61.95, 61.82)
MRIP	GBM	0.99 (0.09)	0.06 (-0.05, 0.21)	0.10 (-61.85, 61.38)
MRIP	NSCLC	1.00 (0.06)	0.05 (-0.06, 0.15)	0.02 (-61.94, 62.08)
MRIP	OFTPP	1.00 (0.06)	0.05 (-0.05, 0.15)	0.09 (-61.78, 61.94)
MRIP	RCC	1.00 (0.06)	0.05 (-0.08, 0.17)	0.02 (-62.08, 61.97)

Table E12: Mixture probability estimates (as mean and standard deviation), and informative and vague mixture component estimates (as median and 95% credible interval), for slopes from application of surrogacy models to data at present-day. MCIP - Mixed common and independent parameters, MRIP - Mixed random and independent parameters, BC - Breast cancer, CC - Cervical cancer, CRC - Colorectal cancer, GBM - Glioblastoma, NSCLC - Non-small cell lung cancer, OFTPP - Ovarian, fallopian tube, and primary peritoneal, RCC - Renal cell carcinoma.

Model	Indication	Probability	Informative	Vague
MCIP	BC	1.00 (0.06)	0.42 (0.22, 0.63)	0.17 (-62.07, 62.29)
MCIP	CC	0.96 (0.20)	0.42 (0.22, 0.63)	0.61 (-61.25, 61.56)
MCIP	CRC	0.92 (0.26)	0.42 (0.22, 0.63)	0.54 (-60.83, 60.93)
MCIP	GBM	0.98 (0.14)	0.42 (0.22, 0.63)	0.18 (-61.74, 61.74)
MCIP	NSCLC	0.99 (0.08)	0.42 (0.22, 0.63)	0.32 (-61.88, 61.93)
MCIP	OFTPP	0.98 (0.15)	0.42 (0.22, 0.63)	0.23 (-61.61, 61.29)
MCIP	RCC	1.00 (0.07)	0.42 (0.22, 0.63)	0.17 (-62.14, 62.01)
MRIP	BC	0.99 (0.08)	0.42 (0.15, 0.69)	0.26 (-61.93, 62.03)
MRIP	CC	0.97 (0.17)	0.47 (0.11, 0.91)	0.53 (-61.42, 61.47)
MRIP	CRC	0.98 (0.14)	0.52 (0.27, 0.74)	0.47 (-61.77, 61.85)
MRIP	GBM	0.98 (0.12)	0.35 (0.02, 0.63)	0.15 (-61.60, 61.96)
MRIP	NSCLC	0.99 (0.09)	0.47 (0.22, 0.71)	0.21 (-62.07, 61.96)
MRIP	OFTPP	0.99 (0.12)	0.34 (0.10, 0.59)	0.20 (-61.65, 61.76)
MRIP	RCC	0.99 (0.10)	0.43 (0.12, 0.73)	0.22 (-61.70, 61.56)

Table E13: Mixture probability estimates (as mean and standard deviation), and informative and vague mixture component estimates (as median and 95% credible interval), for conditional variances from application of surrogacy models to data at present-day. MCIP - Mixed common and independent parameters, MRIP - Mixed random and independent parameters, BC - Breast cancer, CC - Cervical cancer, CRC - Colorectal cancer, GBM - Glioblastoma, NSCLC - Non-small cell lung cancer, OFTPP - Ovarian, fallopian tube, and primary peritoneal, RCC - Renal cell carcinoma.

Model	Indication	Probability	Informative	Vague
MCIP	BC	0.82 (0.38)	0.05 (0.00, 0.13)	0.26 (0.01, 1.08)
MCIP	CC	0.58 (0.49)	0.05 (0.00, 0.13)	0.28 (0.01, 1.06)
MCIP	CRC	0.82 (0.38)	0.05 (0.00, 0.13)	0.25 (0.01, 1.08)
MCIP	GBM	0.57 (0.50)	0.05 (0.00, 0.13)	0.24 (0.02, 1.02)
MCIP	NSCLC	0.81 (0.39)	0.05 (0.00, 0.13)	0.25 (0.01, 1.08)
MCIP	OFTPP	0.78 (0.41)	0.05 (0.00, 0.13)	0.25 (0.01, 1.07)
MCIP	RCC	0.78 (0.41)	0.05 (0.00, 0.13)	0.26 (0.01, 1.07)
MRIP	BC	0.79 (0.40)	0.04 (0.00, 0.16)	0.24 (0.01, 1.08)
MRIP	CC	0.60 (0.49)	0.05 (0.00, 0.22)	0.29 (0.01, 1.06)
MRIP	CRC	0.82 (0.39)	0.04 (0.00, 0.15)	0.25 (0.01, 1.08)
MRIP	GBM	0.68 (0.46)	0.06 (0.00, 0.21)	0.24 (0.01, 1.05)
MRIP	NSCLC	0.79 (0.41)	0.04 (0.00, 0.17)	0.24 (0.01, 1.07)
MRIP	OFTPP	0.80 (0.40)	0.04 (0.00, 0.17)	0.25 (0.01, 1.07)
MRIP	RCC	0.75 (0.43)	0.04 (0.00, 0.19)	0.25 (0.01, 1.06)

E.2 TA178 (renal)

E.2.1 Pooled OS and PFS estimates

Table E14: Median and 95% credible interval estimates for overall survival using data available at the time of NICE TA178. IP - Independent parameters, CP - Common parameters, MCIP - Mixed common and independent parameters, RP - Random parameters, MRIP - Mixed random and independent parameters, BC - Breast cancer, CC - Cervical cancer, CRC - Colorectal cancer, GBM - Glioblastoma, NSCLC - Non-small cell lung cancer, OFTPP - Ovarian, fallopian tube, and primary peritoneal, RCC - Renal cell carcinoma.

Indication	IP	CP	MCIP	RP	MRIP
CRC	-0.23 (-0.41, -0.06)	-0.16 (-0.25, -0.06)	-0.16 (-0.25, -0.06)	-0.19 (-0.33, -0.06)	-0.19 (-0.33, -0.06)
BC	-0.10 (-0.29, 0.10)	-0.16 (-0.25, -0.06)	-0.16 (-0.25, -0.06)	-0.13 (-0.26, 0.02)	-0.13 (-0.26, 0.01)
NSCLC	-0.11 (-0.73, 0.52)	-0.16 (-0.25, -0.06)	-0.16 (-0.25, -0.06)	-0.14 (-0.37, 0.10)	-0.14 (-0.35, 0.08)
RCC	-0.15 (-0.65, 0.35)	-0.16 (-0.25, -0.06)	-0.16 (-0.25, -0.06)	-0.15 (-0.34, 0.04)	-0.15 (-0.33, 0.03)
All Indications		-0.16 (-0.25, -0.06)	-0.16 (-0.25, -0.06)	-0.16 (-0.38, 0.08)	-0.15 (-0.34, 0.04)
New Indication				-0.16 (-0.61, 0.31)	-0.15 (-0.52, 0.23)

Table E15: Median and 95% credible interval estimates for progression-free survival using data available at the time of NICE TA178. IP - Independent parameters, CP - Common parameters, MCIP - Mixed common and independent parameters, RP - Random parameters, MRIP - Mixed random and independent parameters, BC - Breast cancer, CC - Cervical cancer, CRC - Colorectal cancer, GBM - Glioblastoma, NSCLC - Non-small cell lung cancer, OFTPP - Ovarian, fallopian tube, and primary peritoneal, RCC - Renal cell carcinoma.

Indication	IP	CP	MCIP	RP	MRIP
CRC	-0.48 (-0.76, -0.26)	-0.40 (-0.52, -0.30)	-0.40 (-0.52, -0.29)	-0.43 (-0.65, -0.28)	-0.43 (-0.64, -0.28)
BC	-0.34 (-0.54, -0.15)	-0.40 (-0.52, -0.30)	-0.40 (-0.52, -0.29)	-0.37 (-0.53, -0.22)	-0.37 (-0.53, -0.22)
NSCLC	-0.29 (-0.91, 0.33)	-0.40 (-0.52, -0.30)	-0.40 (-0.52, -0.29)	-0.37 (-0.63, -0.09)	-0.37 (-0.62, -0.11)
RCC	-0.50 (-1.01, -0.15)	-0.40 (-0.52, -0.30)	-0.40 (-0.52, -0.29)	-0.44 (-0.70, -0.25)	-0.44 (-0.68, -0.26)
All Indications		-0.40 (-0.52, -0.30)	-0.40 (-0.52, -0.29)	-0.40 (-0.69, -0.13)	-0.40 (-0.64, -0.18)
New Indication				-0.40 (-0.98, 0.17)	-0.40 (-0.85, 0.04)

E.2.2 Predicted OS estimates

Table E16: Median and 95% credible interval estimates for predicted overall survival, from entering the independent parameters (IP) progression-free survival estimate into the surrogacy models, using data available at the time of NICE TA178. IP - Independent parameters, CP - Common parameters, MCIP - Mixed common and independent parameters, RP - Random parameters, MRIP - Mixed random and independent parameters, BC - Breast cancer, CC - Cervical cancer, CRC - Colorectal cancer, GBM - Glioblastoma, NSCLC - Non-small cell lung cancer, OFTPP - Ovarian, fallopian tube, and primary peritoneal, RCC - Renal cell carcinoma.

Indication	IP	CP	MCIP	RP	MRIP
RCC	-0.16 (-8.63, 8.18)	-0.15 (-14.79, 14.26)	-0.16 (-16.27, 15.96)	-0.14 (-13.51, 13.31)	-0.15 (-15.45, 15.08)

Table E17: Median and 95% credible interval estimates for predicted overall survival, from entering the model-specific progression-free survival estimate into the corresponding surrogacy models, using data available at the time of NICE TA178. IP - Independent parameters, CP - Common parameters, MCIP - Mixed common and independent parameters, RP - Random parameters, MRIP - Mixed random and independent parameters, BC - Breast cancer, CC - Cervical cancer, CRC - Colorectal cancer, GBM - Glioblastoma, NSCLC - Non-small cell lung cancer, OFTPP - Ovarian, fallopian tube, and primary peritoneal, RCC - Renal cell carcinoma.

Indication	IP	CP	MCIP	RP	MRIP
RCC	-0.16 (-8.63, 8.18)	-0.16 (-0.59, 0.28)	-0.17 (-0.68, 0.30)	-0.12 (-0.51, 0.28)	-0.14 (-0.64, 0.32)

E.2.3 Deviance information criterion (DIC)

Table E18: Residual deviance, deviance, number of effective parameters, and deviance information criterian (DIC) from application of models to data on overall survival at the time of NICE TA178. *Based on 13 data points. IP - Independent parameters, CP - Common parameter, MCIP - Mixed common and independent parameters, RP - Random parameters, MRIP - Mixed random and independent parameters

Model	Residual deviance	Deviance	p_D	DIC
IP	11.13	-16.92	8.99	-7.92
CP	10.92	-17.13	7.56	-9.57
MCIP	10.94	-17.11	7.59	-9.52
RP	10.87	-17.18	8.23	-8.95
MRIP	10.84	-15.82	8.16	-7.66

Table E19: Residual deviance, deviance, number of effective parameters, and deviance information criterian (DIC) from application of models to data on progression-free survival at the time of NICE TA178. *Based on 17 data points. IP - Independent parameters, CP - Common parameter, MCIP - Mixed common and independent parameters, RP - Random parameters, MRIP - Mixed random and independent parameters

Model	Residual deviance	Deviance	p_D	DIC
IP	18.30	-15.98	13.56	-2.42
CP	19.48	-14.80	12.37	-2.43
MCIP	19.50	-14.78	12.42	-2.36
RP	18.81	-15.47	12.91	-2.56
MRIP	18.87	-14.02	12.86	-1.16

E.2.4 Mixture probabilities

Table E20: Mixture probability estimates (as mean and standard deviation), and informative and vague mixture component estimates (as median and 95% credible interval), from application of models to data on overall survival at the time of NICE TA178. MCIP - Mixed common and independent parameters, MRIP - Mixed random and independent parameters, BC - Breast cancer, CC - Cervical cancer, CRC - Colorectal cancer, GBM - Glioblastoma, NSCLC - Non-small cell lung cancer, OFTPP - Ovarian, fallopian tube, and primary peritoneal, RCC - Renal cell carcinoma.

Model	Indication	Probability	Informative	Vague
MCIP	Breast	0.99 (0.07)	-0.16 (-0.25, -0.06)	-0.08 (-61.86, 61.60)
MCIP	Colorectal	0.99 (0.09)	-0.16 (-0.25, -0.06)	-0.12 (-61.93, 62.00)
MCIP	NSCLC	0.99 (0.08)	-0.16 (-0.25, -0.06)	-0.03 (-62.07, 61.98)
MCIP	Renal	0.99 (0.07)	-0.16 (-0.25, -0.06)	-0.02 (-61.95, 61.66)
MRIP	Breast	0.99 (0.08)	-0.13 (-0.26, 0.01)	-0.08 (-61.97, 61.69)
MRIP	Colorectal	0.99 (0.08)	-0.19 (-0.33, -0.05)	-0.13 (-61.90, 61.54)
MRIP	NSCLC	0.99 (0.09)	-0.14 (-0.35, 0.08)	-0.05 (-61.84, 61.86)
MRIP	Renal	0.99 (0.09)	-0.15 (-0.34, 0.03)	-0.07 (-62.01, 62.01)

Table E21: Mixture probability estimates (as mean and standard deviation), and informative and vague mixture component estimates (as median and 95% credible interval), from application of models to data on progression-free survival at the time of NICE TA178. MCIP - Mixed common and independent parameters, MRIP - Mixed random and independent parameters, BC - Breast cancer, CC - Cervical cancer, CRC - Colorectal cancer, GBM - Glioblastoma, NSCLC - Non-small cell lung cancer, OFTPP - Ovarian, fallopian tube, and primary peritoneal, RCC - Renal cell carcinoma.

Model	Indication	Probability	Informative	Vague
MCIP	Breast	1.00 (0.07)	-0.40 (-0.52, -0.29)	-0.22 (-62.13, 61.50)
MCIP	Colorectal	0.99 (0.08)	-0.40 (-0.52, -0.29)	-0.24 (-62.03, 62.10)
MCIP	NSCLC	0.99 (0.10)	-0.40 (-0.52, -0.29)	-0.15 (-61.99, 61.80)
MCIP	Renal	0.99 (0.09)	-0.40 (-0.52, -0.29)	-0.27 (-62.29, 61.98)
MRIP	Breast	0.99 (0.09)	-0.37 (-0.53, -0.22)	-0.14 (-61.44, 61.65)
MRIP	Colorectal	0.99 (0.08)	-0.43 (-0.64, -0.28)	-0.15 (-62.20, 61.48)
MRIP	NSCLC	0.99 (0.11)	-0.37 (-0.62, -0.11)	-0.12 (-62.08, 61.79)
MRIP	Renal	0.99 (0.10)	-0.44 (-0.68, -0.25)	-0.41 (-61.91, 62.15)

E.3 TA285 (ovarian)

E.3.1 Pooled OS and PFS estimates

Table E22: Median and 95% credible interval estimates for overall survival using data available at the time of NICE TA285. IP - Independent parameters, CP - Common parameters, MCIP - Mixed common and independent parameters, RP - Random parameters, MRIP - Mixed random and independent parameters, BC - Breast cancer, CC - Cervical cancer, CRC - Colorectal cancer, GBM - Glioblastoma, NSCLC - Non-small cell lung cancer, OFTPP - Ovarian, fallopian tube, and primary peritoneal, RCC - Renal cell carcinoma.

Indication	IP	CP	MCIP	RP	MRIP
CRC	-0.19 (-0.33, -0.07)	-0.13 (-0.20, -0.07)	-0.13 (-0.20, -0.07)	-0.16 (-0.26, -0.07)	-0.16 (-0.26, -0.07)
RCC	-0.15 (-0.65, 0.34)	-0.13 (-0.20, -0.07)	-0.13 (-0.20, -0.07)	-0.14 (-0.27, -0.01)	-0.14 (-0.27, -0.01)
BC	-0.12 (-0.31, 0.04)	-0.13 (-0.20, -0.07)	-0.13 (-0.20, -0.07)	-0.13 (-0.23, -0.02)	-0.13 (-0.23, -0.03)
NSCLC	-0.18 (-0.63, 0.25)	-0.13 (-0.20, -0.07)	-0.13 (-0.20, -0.07)	-0.14 (-0.29, -0.01)	-0.14 (-0.29, -0.01)
CC	-0.34 (-1.46, 0.78)	-0.13 (-0.20, -0.07)	-0.13 (-0.21, -0.07)	-0.14 (-0.36, 0.02)	-0.14 (-0.37, 0.02)
GBM	-0.02 (-0.62, 0.61)	-0.13 (-0.20, -0.07)	-0.13 (-0.20, -0.07)	-0.12 (-0.26, 0.06)	-0.12 (-0.26, 0.06)
OFTPP	-0.06 (-0.25, 0.12)	-0.13 (-0.20, -0.07)	-0.13 (-0.20, -0.07)	-0.11 (-0.21, 0.00)	-0.11 (-0.21, 0.01)
All Indications		-0.13 (-0.20, -0.07)	-0.13 (-0.20, -0.07)	-0.13 (-0.24, -0.04)	-0.13 (-0.23, -0.04)
New Indication				-0.13 (-0.34, 0.07)	-0.13 (-0.34, 0.06)

Table E23: Median and 95% credible interval estimates for progression-free survival using data available at the time of NICE TA285. IP - Independent parameters, CP - Common parameters, MCIP - Mixed common and independent parameters, RP - Random parameters, MRIP - Mixed random and independent parameters, BC - Breast cancer, CC - Cervical cancer, CRC - Colorectal cancer, GBM - Glioblastoma, NSCLC - Non-small cell lung cancer, OFTPP - Ovarian, fallopian tube, and primary peritoneal, RCC - Renal cell carcinoma.

Indication	IP	CP	MCIP	RP	MRIP
CRC	-0.47 (-0.67, -0.30)	-0.38 (-0.47, -0.31)	-0.38 (-0.48, -0.30)	-0.42 (-0.58, -0.30)	-0.42 (-0.58, -0.30)
RCC	-0.50 (-1.01, -0.15)	-0.38 (-0.47, -0.31)	-0.38 (-0.48, -0.30)	-0.42 (-0.62, -0.28)	-0.42 (-0.62, -0.28)
BC	-0.30 (-0.42, -0.18)	-0.38 (-0.47, -0.31)	-0.38 (-0.47, -0.30)	-0.34 (-0.45, -0.23)	-0.34 (-0.45, -0.23)
NSCLC	-0.47 (-1.08, 0.11)	-0.38 (-0.47, -0.31)	-0.38 (-0.48, -0.30)	-0.40 (-0.63, -0.22)	-0.40 (-0.63, -0.22)
CC	-0.40 (-1.50, 0.69)	-0.38 (-0.47, -0.31)	-0.38 (-0.48, -0.30)	-0.39 (-0.61, -0.19)	-0.39 (-0.62, -0.19)
GBM	-0.35 (-0.93, 0.24)	-0.38 (-0.47, -0.31)	-0.38 (-0.48, -0.30)	-0.38 (-0.56, -0.21)	-0.38 (-0.56, -0.21)
OFTPP	-0.43 (-0.90, 0.02)	-0.38 (-0.47, -0.31)	-0.38 (-0.48, -0.30)	-0.40 (-0.61, -0.22)	-0.40 (-0.61, -0.23)
All Indications		-0.38 (-0.47, -0.31)	-0.38 (-0.48, -0.30)	-0.39 (-0.54, -0.28)	-0.39 (-0.53, -0.28)
New Indication				-0.39 (-0.68, -0.14)	-0.39 (-0.67, -0.15)

E.3.2 Predicted OS estimates

Table E24: Median and 95% credible interval estimates for predicted overall survival, from entering the independent parameters (IP) progression-free survival estimate into the surrogacy models, using data available at the time of NICE TA285. IP - Independent parameters, CP - Common parameters, MCIP - Mixed common and independent parameters, RP - Random parameters, MRIP - Mixed random and independent parameters, BC - Breast cancer, CC - Cervical cancer, CRC - Colorectal cancer, GBM - Glioblastoma, NSCLC - Non-small cell lung cancer, OFTPP - Ovarian, fallopian tube, and primary peritoneal, RCC - Renal cell carcinoma.

Indication	IP	CP	MCIP	RP	MRIP
OFTPP	-0.07 (-0.39, 0.27)	-0.07 (-0.48, 0.37)	-0.07 (-0.49, 0.38)	-0.06 (-0.46, 0.35)	-0.06 (-0.45, 0.33)

Table E25: Median and 95% credible interval estimates for predicted overall survival, from entering the model-specific progression-free survival estimate into the corresponding surrogacy models, using data available at the time of NICE TA285. IP - Independent parameters, CP - Common parameters, MCIP - Mixed common and independent parameters, RP - Random parameters, MRIP - Mixed random and independent parameters, BC - Breast cancer, CC - Cervical cancer, CRC - Colorectal cancer, GBM - Glioblastoma, NSCLC - Non-small cell lung cancer, OFTPP - Ovarian, fallopian tube, and primary peritoneal, RCC - Renal cell carcinoma.

Indication	IP	CP	MCIP	RP	MRIP
OFTPP	-0.07 (-0.39, 0.27)	-0.13 (-0.47, 0.21)	-0.13 (-0.51, 0.23)	-0.06 (-0.33, 0.18)	-0.05 (-0.31, 0.18)

E.3.3 Deviance information criterion (DIC)

Table E26: Residual deviance, deviance, number of effective parameters, and deviance information criterian (DIC) from application of models to data on overall survival at the time of NICE TA285. *Based on 27 data points. IP - Independent parameters, CP - Common parameter, MCIP - Mixed common and independent parameters, RP - Random parameters, MRIP - Mixed random and independent parameters

Model	Residual deviance	Deviance	p_D	DIC
IP	26.94	-30.43	18.83	-11.59
CP	27.60	-29.77	16.02	-13.74
MCIP	27.57	-29.80	15.99	-13.81
RP	27.12	-30.25	16.78	-13.47
MRIP	27.12	-28.86	16.75	-12.11

Table E27: Residual deviance, deviance, number of effective parameters, and deviance information criterian (DIC) from application of models to data on progression-free survival at the time of NICE TA285. *Based on 32 data points. IP - Independent parameters, CP - Common parameter, MCIP - Mixed common and independent parameters, RP - Random parameters, MRIP - Mixed random and independent parameters

Model	Residual deviance	Deviance	p_D	DIC
IP	33.27	-36.97	24.75	-12.22
CP	34.90	-35.33	23.90	-11.43
MCIP	35.03	-35.21	23.89	-11.31
RP	34.13	-36.11	23.86	-12.25
MRIP	34.10	-34.75	23.87	-10.88

E.3.4 Mixture probabilities

Table E28: Mixture probability estimates (as mean and standard deviation), and informative and vague mixture component estimates (as median and 95% credible interval), from application of models to data on overall survival at the time of NICE TA285. MCIP - Mixed common and independent parameters, MRIP - Mixed random and independent parameters, BC - Breast cancer, CC - Cervical cancer, CRC - Colorectal cancer, GBM - Glioblastoma, NSCLC - Non-small cell lung cancer, OFTPP - Ovarian, fallopian tube, and primary peritoneal, RCC - Renal cell carcinoma.

Model	Indication	Probability	Informative	Vague
MCIP	Breast	1.00 (0.06)	-0.13 (-0.20, -0.07)	-0.04 (-62.02, 61.96)
MCIP	Cervical	0.99 (0.12)	-0.13 (-0.20, -0.07)	-0.26 (-61.67, 61.68)
MCIP	Colorectal	0.99 (0.07)	-0.13 (-0.20, -0.07)	-0.10 (-61.92, 62.13)
MCIP	Glioblastoma	0.99 (0.10)	-0.13 (-0.20, -0.07)	-0.03 (-61.37, 62.03)
MCIP	NSCLC	0.99 (0.07)	-0.13 (-0.20, -0.07)	0.03 (-62.02, 61.99)
MCIP	OFTPP	0.99 (0.08)	-0.13 (-0.20, -0.07)	-0.04 (-61.74, 61.76)
MCIP	Renal	0.99 (0.07)	-0.13 (-0.20, -0.07)	-0.04 (-61.78, 61.72)
MRIP	Breast	1.00 (0.06)	-0.13 (-0.23, -0.03)	-0.11 (-62.19, 61.87)
MRIP	Cervical	0.99 (0.12)	-0.14 (-0.35, 0.01)	-0.21 (-61.91, 61.77)
MRIP	Colorectal	0.99 (0.07)	-0.15 (-0.26, -0.07)	-0.12 (-61.81, 62.33)
MRIP	Glioblastoma	0.99 (0.10)	-0.12 (-0.26, 0.05)	-0.01 (-61.88, 61.65)
MRIP	NSCLC	1.00 (0.07)	-0.14 (-0.29, -0.01)	-0.16 (-62.12, 61.89)
MRIP	OFTPP	0.99 (0.08)	-0.11 (-0.22, 0.00)	-0.07 (-62.13, 62.00)
MRIP	Renal	1.00 (0.07)	-0.14 (-0.27, -0.01)	-0.12 (-61.84, 61.97)

Table E29: Mixture probability estimates (as mean and standard deviation), and informative and vague mixture component estimates (as median and 95% credible interval), from application of models to data on progression-free survival at the time of NICE TA285. MCIP - Mixed common and independent parameters, MRIP - Mixed random and independent parameters, BC - Breast cancer, CC - Cervical cancer, CRC - Colorectal cancer, GBM - Glioblastoma, NSCLC - Non-small cell lung cancer, OFTPP - Ovarian, fallopian tube, and primary peritoneal, RCC - Renal cell carcinoma.

Model	Indication	Probability	Informative	Vague
MCIP	Breast	0.98 (0.13)	-0.38 (-0.48, -0.30)	-0.28 (-61.64, 61.64)
MCIP	Cervical	0.99 (0.10)	-0.38 (-0.48, -0.30)	-0.06 (-61.88, 62.08)
MCIP	Colorectal	0.99 (0.08)	-0.38 (-0.48, -0.30)	-0.21 (-62.00, 61.83)
MCIP	Glioblastoma	0.99 (0.07)	-0.38 (-0.48, -0.30)	-0.20 (-61.44, 62.06)
MCIP	NSCLC	0.99 (0.08)	-0.38 (-0.48, -0.30)	-0.18 (-61.85, 61.95)
MCIP	OFTPP	0.99 (0.08)	-0.38 (-0.48, -0.30)	-0.15 (-61.77, 61.92)
MCIP	Renal	0.99 (0.10)	-0.38 (-0.48, -0.30)	-0.42 (-61.98, 62.03)
MRIP	Breast	0.99 (0.08)	-0.34 (-0.46, -0.23)	-0.15 (-62.29, 61.54)
MRIP	Cervical	0.99 (0.10)	-0.39 (-0.61, -0.20)	-0.28 (-61.98, 62.06)
MRIP	Colorectal	0.99 (0.08)	-0.42 (-0.57, -0.30)	-0.18 (-61.84, 62.22)
MRIP	Glioblastoma	0.99 (0.08)	-0.38 (-0.56, -0.21)	-0.23 (-62.05, 61.88)
MRIP	NSCLC	0.99 (0.09)	-0.40 (-0.63, -0.22)	-0.24 (-61.90, 61.95)
MRIP	OFTPP	0.99 (0.08)	-0.40 (-0.60, -0.23)	-0.23 (-61.96, 62.09)
MRIP	Renal	0.99 (0.09)	-0.42 (-0.61, -0.28)	-0.25 (-61.72, 62.13)

E.4 Surrogate endpoint cross validation

Table E30: Median and 95% credible interval estimates for overall survival from applying leave-one-out cross validation to evaluate surrogacy using trial data at present day. IP - Independent parameters, CP - Common parameter, MCIP - Mixed common and independent parameters, RP - Random parameters, MRIP - Mixed random and independent parameters, NSCLC - Non-small cell lung cancer, OFTPP - Ovarian, fallopian tube, and primary peritoneal

Trial	Observed	IP	CP	MCIP	RP	MRIP
**Breast**						
E2100	-0.13 (-0.30, 0.05)	-0.22 (-0.75, 0.30)	-0.18 (-0.43, 0.08)	-0.18 (-0.45, 0.08)	-0.18 (-0.47, 0.10)	-0.19 (-0.48, 0.11)
RIBBON-1	-0.16 (-0.46, 0.13)	-0.12 (-0.55, 0.32)	-0.11 (-0.44, 0.22)	-0.12 (-0.45, 0.22)	-0.11 (-0.45, 0.23)	-0.11 (-0.46, 0.24)
RIBBON-1	0.03 (-0.26, 0.32)	-0.12 (-0.55, 0.32)	-0.11 (-0.44, 0.22)	-0.12 (-0.45, 0.22)	-0.11 (-0.45, 0.23)	-0.11 (-0.46, 0.24)
RIBBON-2	-0.11 (-0.34, 0.13)	-0.06 (-0.48, 0.37)	-0.06 (-0.34, 0.22)	-0.06 (-0.35, 0.22)	-0.06 (-0.34, 0.23)	-0.06 (-0.36, 0.24)
AVADO	0.03 (-0.29, 0.35)	-0.09 (-0.54, 0.36)	-0.07 (-0.42, 0.28)	-0.07 (-0.43, 0.29)	-0.07 (-0.43, 0.29)	-0.07 (-0.44, 0.30)
SUN 1094	-0.60 (-1.05, -0.15)	-0.14 (-0.67, 0.40)	-0.16 (-0.65, 0.32)	-0.17 (-0.66, 0.32)	-0.15 (-0.63, 0.34)	-0.14 (-0.63, 0.34)
LEA	-0.14 (-0.55, 0.27)	-0.01 (-0.59, 0.56)	-0.04 (-0.48, 0.41)	-0.04 (-0.48, 0.41)	-0.03 (-0.48, 0.42)	-0.03 (-0.48, 0.42)
MERiDiAN	-0.21 (-0.50, 0.07)	-0.12 (-0.57, 0.33)	-0.12 (-0.45, 0.21)	-0.12 (-0.46, 0.22)	-0.11 (-0.45, 0.23)	-0.11 (-0.46, 0.24)
TANIA	-0.04 (-0.27, 0.19)	-0.09 (-0.51, 0.32)	-0.08 (-0.36, 0.19)	-0.08 (-0.37, 0.20)	-0.08 (-0.37, 0.21)	-0.08 (-0.38, 0.22)
E1105	0.09 (-0.50, 0.67)	-0.10 (-0.85, 0.64)	-0.09 (-0.73, 0.55)	-0.09 (-0.74, 0.55)	-0.09 (-0.73, 0.55)	-0.09 (-0.74, 0.55)
**Cervical**						
GOG240	-0.26 (-0.47, -0.05)	0.00 (-0.21, 0.21)	-0.12 (-0.37, 0.14)	3.36 (-22.48, 29.19)	-0.11 (-0.46, 0.25)	-1.81 (-43.43, 39.81)
**Colorectal**						
AVF2107g	-0.42 (-0.66, -0.18)	-0.28 (-0.65, 0.09)	-0.20 (-0.51, 0.10)	-0.20 (-0.51, 0.10)	-0.20 (-0.51, 0.10)	-0.20 (-0.51, 0.10)
AVF2192g	-0.24 (-0.57, 0.10)	-0.38 (-0.84, 0.09)	-0.25 (-0.65, 0.15)	-0.25 (-0.65, 0.15)	-0.25 (-0.65, 0.15)	-0.25 (-0.65, 0.15)
E3200	-0.29 (-0.46, -0.11)	-0.23 (-0.53, 0.08)	-0.16 (-0.39, 0.07)	-0.16 (-0.39, 0.07)	-0.16 (-0.39, 0.07)	-0.16 (-0.39, 0.07)
NO16966	-0.12 (-0.27, 0.04)	-0.02 (-0.31, 0.26)	-0.03 (-0.23, 0.18)	-0.03 (-0.23, 0.18)	-0.03 (-0.23, 0.18)	-0.03 (-0.23, 0.18)
MAX	-0.13 (-0.38, 0.13)	-0.24 (-0.59, 0.11)	-0.16 (-0.46, 0.14)	-0.16 (-0.46, 0.14)	-0.16 (-0.46, 0.14)	-0.16 (-0.46, 0.14)
HORIZON III	0.05 (-0.10, 0.20)	-0.06 (-0.36, 0.25)	-0.01 (-0.21, 0.20)	-0.01 (-0.21, 0.20)	-0.01 (-0.21, 0.20)	-0.01 (-0.21, 0.20)
ARTIST	-0.48 (-0.90, -0.06)	-0.42 (-0.95, 0.12)	-0.29 (-0.76, 0.17)	-0.29 (-0.76, 0.17)	-0.29 (-0.76, 0.17)	-0.29 (-0.76, 0.17)
ML18147	-0.19 (-0.34, -0.03)	-0.18 (-0.46, 0.10)	-0.12 (-0.33, 0.08)	-0.12 (-0.33, 0.08)	-0.12 (-0.33, 0.08)	-0.12 (-0.33, 0.08)
AVEX	-0.24 (-0.56, 0.09)	-0.33 (-0.75, 0.09)	-0.23 (-0.59, 0.14)	-0.23 (-0.59, 0.14)	-0.23 (-0.59, 0.14)	-0.23 (-0.59, 0.14)
**Glioblastoma**						
RTOG0825	0.12 (-0.07, 0.32)	0.00 (-0.19, 0.19)	-0.07 (-0.30, 0.16)	-0.06 (-0.48, 0.35)	-0.03 (-0.30, 0.24)	-0.03 (-0.45, 0.38)
AvaGlio	-0.13 (-0.27, 0.02)	0.00 (-0.15, 0.15)	-0.15 (-0.35, 0.06)	-0.14 (-0.73, 0.46)	-0.04 (-0.32, 0.23)	-0.06 (-0.54, 0.42)
EORTC26101	-0.05 (-0.30, 0.19)	0.00 (-0.25, 0.25)	-0.28 (-0.57, 0.02)	-0.28 (-0.72, 0.16)	-0.23 (-0.59, 0.13)	-0.23 (-0.72, 0.25)

**NSCLC**						
E4599	-0.24 (-0.39, -0.08)	-0.10 (-0.50, 0.31)	-0.13 (-0.33, 0.08)	-0.12 (-0.35, 0.10)	-0.12 (-0.35, 0.11)	-0.12 (-0.37, 0.13)
AVAiL	0.03 (-0.15, 0.21)	-0.02 (-0.59, 0.54)	-0.03 (-0.26, 0.19)	-0.03 (-0.29, 0.22)	-0.05 (-0.30, 0.20)	-0.05 (-0.32, 0.22)
BEYOND	-0.39 (-0.70, -0.08)	-0.51 (-1.48, 0.46)	-0.33 (-0.70, 0.04)	-0.35 (-0.74, 0.05)	-0.39 (-0.82, 0.05)	-0.39 (-0.84, 0.06)
JO25567	-0.21 (-0.63, 0.21)	-0.29 (-0.95, 0.37)	-0.23 (-0.70, 0.24)	-0.24 (-0.72, 0.24)	-0.27 (-0.76, 0.23)	-0.26 (-0.77, 0.24)
IMpower150	0.08 (-0.51, 0.67)	0.02 (-0.78, 0.81)	-0.01 (-0.64, 0.62)	-0.01 (-0.65, 0.63)	-0.02 (-0.66, 0.63)	-0.02 (-0.67, 0.64)
NEJ026	0.01 (-0.38, 0.40)	-0.06 (-0.67, 0.55)	-0.07 (-0.50, 0.36)	-0.07 (-0.51, 0.37)	-0.08 (-0.52, 0.36)	-0.08 (-0.54, 0.38)
**OFTPP**						
GOG218	-0.04 (-0.17, 0.08)	-0.04 (-0.47, 0.39)	-0.07 (-0.26, 0.11)	-0.07 (-0.32, 0.18)	-0.04 (-0.25, 0.18)	-0.04 (-0.29, 0.21)
ICON7	-0.01 (-0.16, 0.14)	0.03 (-0.50, 0.56)	0.02 (-0.20, 0.23)	0.02 (-0.24, 0.28)	0.04 (-0.19, 0.27)	0.04 (-0.22, 0.30)
OCEANS	-0.05 (-0.26, 0.16)	-0.23 (-0.66, 0.21)	-0.29 (-0.56, -0.02)	-0.30 (-0.58, -0.03)	-0.26 (-0.54, 0.02)	-0.26 (-0.55, 0.03)
AURELIA	-0.16 (-0.41, 0.08)	-0.15 (-0.67, 0.37)	-0.28 (-0.58, 0.02)	-0.28 (-0.63, 0.06)	-0.21 (-0.53, 0.12)	-0.21 (-0.56, 0.14)
GOG213	-0.19 (-0.38, 0.01)	-0.07 (-0.45, 0.32)	-0.15 (-0.39, 0.09)	-0.15 (-0.44, 0.13)	-0.09 (-0.34, 0.16)	-0.09 (-0.37, 0.18)
mEOC/GOG0241	0.39 (-0.58, 1.35)	0.02 (-1.05, 1.09)	0.08 (-0.97, 1.13)	0.09 (-0.97, 1.14)	0.08 (-0.94, 1.11)	0.09 (-0.95, 1.12)
mEOC/GOG0241	-0.26 (-1.23, 0.71)	0.02 (-1.05, 1.09)	0.08 (-0.97, 1.13)	0.09 (-0.97, 1.14)	0.08 (-0.94, 1.11)	0.09 (-0.95, 1.12)
**Renal**						
AVOREN	-0.15 (-0.33, 0.03)	-0.06 (-11.23, 11.10)	-0.17 (-0.40, 0.07)	-0.17 (-0.61, 0.26)	-0.18 (-0.47, 0.12)	-0.32 (-2.83, 2.20)
CALGB-90206	-0.15 (-0.36, 0.05)	-0.45 (-9.19, 8.29)	-0.13 (-0.37, 0.12)	-0.13 (-0.55, 0.29)	-0.12 (-0.41, 0.17)	-0.12 (-0.62, 0.38)

E.5 Sensitivity analysis: equal mixture weights for surrogacy parameters

E.5.1 Surrogacy parameter estimates

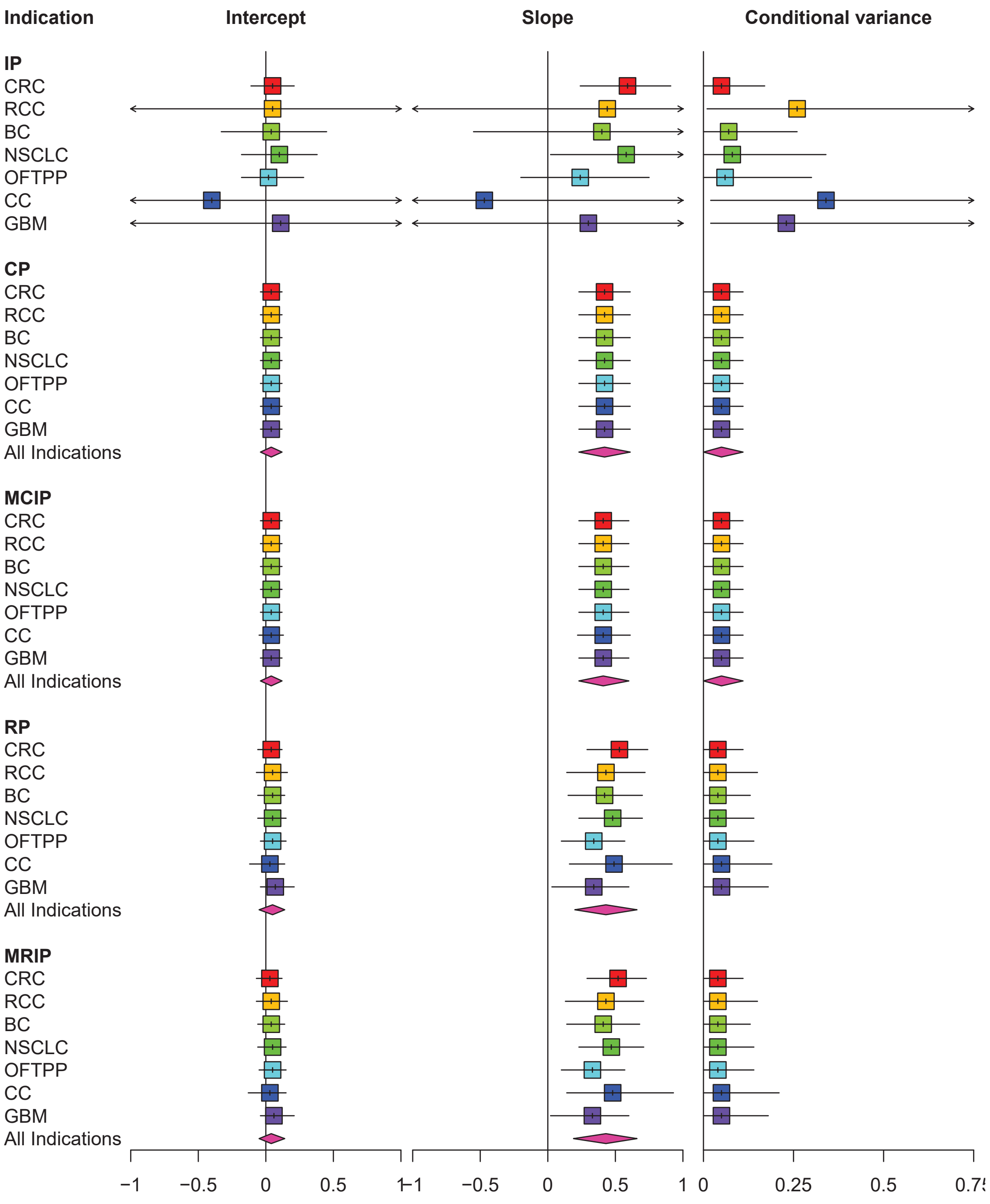

Figure E1: Multi-indication meta-analysis assuming equal mixture weights for surrogacy parameters using data available at present day. IP - independent parameters, CP - common parameter, MCIP - mixed common and independent parameters, RP - random parameters, MRIP - mixed random and independent parameters, CRC - colorectal cancer, BC - breast cancer, NSCLC - non-small cell lung cancer, RCC - renal cell carcinoma, CC - cervical cancer, GBM - glioblastoma, OFTPP - ovarian, fallopian tube, and primary peritoneal.

Table E31: Median and 95% credible interval estimates for intercepts from surrogacy models, including mixture models assuming equal mixture probabilities, using data available at present-day. IP - Independent parameters, CP - Common parameters, MCIP - Mixed common and independent parameters, RP - Random parameters, MRIP - Mixed random and independent parameters, BC - Breast cancer, CC - Cervical cancer, CRC - Colorectal cancer, GBM - Glioblastoma, NSCLC - Non-small cell lung cancer, OFTPP - Ovarian, fallopian tube, and primary peritoneal, RCC - Renal cell carcinoma.

Indication	IP	CP	MCIP	RP	MRIP
CRC	0.05 (-0.11, 0.21)	0.03 (-0.06, 0.13)	0.04 (-0.04, 0.12)	0.03 (-0.09, 0.13)	0.03 (-0.07, 0.12)
RCC	0.03 (-13.71, 17.77)	0.03 (-0.06, 0.13)	0.04 (-0.04, 0.12)	0.03 (-0.11, 0.18)	0.04 (-0.07, 0.16)
BC	0.06 (-0.80, 1.01)	0.03 (-0.06, 0.13)	0.04 (-0.04, 0.12)	0.03 (-0.09, 0.16)	0.04 (-0.06, 0.14)
NSCLC	0.06 (-0.83, 0.93)	0.03 (-0.06, 0.13)	0.04 (-0.04, 0.12)	0.03 (-0.10, 0.16)	0.05 (-0.06, 0.15)
CC	0.47 (-22.10, 26.92)	0.03 (-0.06, 0.13)	0.04 (-0.04, 0.12)	0.02 (-0.18, 0.15)	0.05 (-0.05, 0.15)
GBM	0.48 (-7.14, 12.13)	0.03 (-0.06, 0.13)	0.04 (-0.05, 0.13)	0.05 (-0.08, 0.22)	0.03 (-0.13, 0.15)
OFTPP	-0.05 (-0.52, 0.41)	0.03 (-0.06, 0.13)	0.04 (-0.04, 0.12)	0.03 (-0.09, 0.16)	0.06 (-0.04, 0.21)
All Indications		0.03 (-0.06, 0.13)	0.04 (-0.04, 0.12)	0.03 (-0.08, 0.15)	0.04 (-0.05, 0.14)

Table E32: Median and 95% credible interval estimates for slopes from surrogacy models, including mixture models assuming equal mixture probabilities, using data available at present-day .IP - Independent parameters, CP - Common parameters, MCIP - Mixed common and independent parameters, RP - Random parameters, MRIP - Mixed random and independent parameters, BC - Breast cancer, CC - Cervical cancer, CRC - Colorectal cancer, GBM - Glioblastoma, NSCLC - Non-small cell lung cancer, OFTPP - Ovarian, fallopian tube, and primary peritoneal, RCC - Renal cell carcinoma.

Indication	IP	CP	MCIP	RP	MRIP
CRC	0.59 (0.23, 0.92)	0.42 (0.21, 0.64)	0.41 (0.23, 0.60)	0.50 (0.23, 0.75)	0.52 (0.29, 0.73)
RCC	0.38 (-31.52, 40.46)	0.42 (0.21, 0.64)	0.41 (0.23, 0.60)	0.41 (0.06, 0.75)	0.43 (0.13, 0.71)
BC	0.46 (-1.76, 3.05)	0.42 (0.21, 0.64)	0.41 (0.23, 0.60)	0.40 (0.06, 0.73)	0.41 (0.14, 0.68)
NSCLC	0.54 (-1.12, 2.13)	0.42 (0.21, 0.64)	0.41 (0.23, 0.60)	0.46 (0.16, 0.75)	0.47 (0.23, 0.71)
CC	2.11 (-53.52, 62.53)	0.42 (0.21, 0.64)	0.41 (0.23, 0.60)	0.48 (0.09, 1.00)	0.33 (0.10, 0.57)
GBM	1.42 (-20.10, 34.37)	0.42 (0.21, 0.64)	0.41 (0.22, 0.61)	0.38 (-0.02, 0.74)	0.48 (0.14, 0.93)
OFTPP	0.05 (-0.93, 0.93)	0.42 (0.21, 0.64)	0.41 (0.23, 0.60)	0.30 (-0.02, 0.60)	0.33 (0.02, 0.60)
All Indications		0.42 (0.21, 0.64)	0.41 (0.23, 0.60)	0.42 (0.14, 0.71)	0.43 (0.19, 0.66)

Table E33: Median and 95% credible interval estimates for conditonal variances from surrogacy models, including mixture models assuming equal mixture probabilities, using data available at present-day. IP - Independent parameters, CP - Common parameters, MCIP - Mixed common and independent parameters, RP - Random parameters, MRIP - Mixed random and independent parameters, BC - Breast cancer, CC - Cervical cancer, CRC - Colorectal cancer, GBM - Glioblastoma, NSCLC - Non-small cell lung cancer, OFTPP - Ovarian, fallopian tube, and primary peritoneal, RCC - Renal cell carcinoma.

Indication	IP	CP	MCIP	RP	MRIP
CRC	0.05 (0.00, 0.17)	0.04 (0.00, 0.12)	0.05 (0.00, 0.11)	0.03 (0.00, 0.12)	0.04 (0.00, 0.11)
RCC	0.26 (0.01, 1.01)	0.04 (0.00, 0.12)	0.05 (0.00, 0.11)	0.04 (0.00, 0.17)	0.04 (0.00, 0.15)
BC	0.13 (0.01, 0.48)	0.04 (0.00, 0.12)	0.05 (0.00, 0.11)	0.05 (0.00, 0.17)	0.04 (0.00, 0.13)
NSCLC	0.19 (0.01, 0.85)	0.04 (0.00, 0.12)	0.05 (0.00, 0.11)	0.05 (0.00, 0.17)	0.04 (0.00, 0.14)
CC	0.33 (0.02, 1.12)	0.04 (0.00, 0.12)	0.05 (0.00, 0.11)	0.06 (0.00, 0.22)	0.04 (0.00, 0.14)
GBM	0.30 (0.01, 1.07)	0.04 (0.00, 0.12)	0.05 (0.00, 0.11)	0.06 (0.00, 0.20)	0.05 (0.00, 0.21)
OFTPP	0.12 (0.01, 0.61)	0.04 (0.00, 0.12)	0.05 (0.00, 0.11)	0.05 (0.00, 0.19)	0.05 (0.00, 0.18)
All Indications		0.04 (0.00, 0.12)	0.05 (0.00, 0.11)		

E.5.2 Deviance information criterion (DIC)

Table E34: Residual deviance, deviance, number of effective parameters, and deviance information criterian (DIC) from application of surrogacy models, including mixture models assuming equal mixture weights, to data at present-day. *Based on data points. MCIP - Mixed common and independent parameters, MRIP - Mixed random and independent parameters

Model	Residual deviance	Deviance	p_D	DIC
MCIP	72.93	-109.31	39.37	-69.94
MRIP	71.77	-112.79	42.14	-70.65

E.5.3 Mixture probabilities

Table E35: Mixture probability estimates (as mean and standard deviation), and informative and vague mixture component estimates (as median and 95% credible interval), for intercepts from application of surrogacy models assuming equal mixture probabilities across surrogacy parameters to data at present-day. MCIP - Mixed common and independent parameters, MRIP - Mixed random and independent parameters, NSCLC - Non-small cell lung cancer, OFTPP - Ovarian, fallopian tube, and primary peritoneal.

Model	Indication	Probability	Informative	Vague
MCIP	Breast	1.00 (0.00)	0.04 (-0.04, 0.12)	-0.06 (-62.35, 62.01)
MCIP	Cervical	0.99 (0.08)	0.04 (-0.04, 0.12)	0.06 (-61.73, 61.76)
MCIP	Colorectal	1.00 (0.00)	0.04 (-0.04, 0.12)	-0.01 (-61.93, 61.90)
MCIP	Glioblastoma	1.00 (0.03)	0.04 (-0.04, 0.12)	0.04 (-62.20, 62.16)
MCIP	NSCLC	1.00 (0.00)	0.04 (-0.04, 0.12)	-0.01 (-61.82, 62.07)
MCIP	OFTPP	1.00 (0.00)	0.04 (-0.04, 0.12)	0.02 (-62.11, 61.97)
MCIP	Renal	1.00 (0.01)	0.04 (-0.04, 0.12)	-0.21 (-62.35, 61.95)
MRIP	Breast	1.00 (0.00)	0.04 (-0.06, 0.14)	0.07 (-61.95, 61.72)
MRIP	Cervical	0.99 (0.09)	0.03 (-0.12, 0.14)	0.00 (-61.89, 61.92)
MRIP	Colorectal	1.00 (0.00)	0.03 (-0.07, 0.12)	-0.06 (-62.06, 61.96)
MRIP	Glioblastoma	1.00 (0.01)	0.06 (-0.04, 0.21)	0.20 (-61.81, 62.00)
MRIP	NSCLC	1.00 (0.00)	0.05 (-0.06, 0.15)	-0.02 (-62.13, 62.37)
MRIP	OFTPP	1.00 (0.00)	0.05 (-0.05, 0.15)	0.02 (-61.92, 61.87)
MRIP	Renal	1.00 (0.02)	0.04 (-0.07, 0.16)	-0.04 (-62.16, 62.17)

Table E36: Mixture probability estimates (as mean and standard deviation), and informative and vague mixture component estimates (as median and 95% credible interval), for slopes from application of surrogacy models assuming equal mixture probabilities across surrogacy parameters to data at present-day. MCIP - Mixed common and independent parameters, MRIP - Mixed random and independent parameters, NSCLC - Non-small cell lung cancer, OFTPP - Ovarian, fallopian tube, and primary peritoneal.

Model	Indication	Probability	Informative	Vague
MCIP	Breast	1.00 (0.00)	0.41 (0.23, 0.60)	0.00 (-61.95, 61.94)
MCIP	Cervical	0.99 (0.08)	0.41 (0.23, 0.60)	-0.02 (-61.59, 62.38)
MCIP	Colorectal	1.00 (0.00)	0.41 (0.23, 0.60)	-0.07 (-61.79, 61.92)
MCIP	Glioblastoma	1.00 (0.03)	0.41 (0.23, 0.60)	0.09 (-62.09, 62.04)
MCIP	NSCLC	1.00 (0.00)	0.41 (0.23, 0.60)	-0.10 (-61.96, 62.12)
MCIP	OFTPP	1.00 (0.00)	0.41 (0.23, 0.60)	0.03 (-61.70, 61.99)
MCIP	Renal	1.00 (0.01)	0.41 (0.23, 0.60)	0.19 (-61.86, 61.92)
MRIP	Breast	1.00 (0.00)	0.41 (0.14, 0.68)	-0.14 (-61.80, 61.67)
MRIP	Cervical	0.99 (0.09)	0.48 (0.15, 0.91)	0.20 (-61.87, 62.25)
MRIP	Colorectal	1.00 (0.00)	0.52 (0.29, 0.73)	-0.01 (-61.98, 61.99)
MRIP	Glioblastoma	1.00 (0.01)	0.33 (0.02, 0.60)	0.19 (-61.90, 61.94)
MRIP	NSCLC	1.00 (0.00)	0.47 (0.23, 0.71)	-0.03 (-61.93, 61.92)
MRIP	OFTPP	1.00 (0.00)	0.33 (0.10, 0.57)	0.04 (-61.99, 62.22)
MRIP	Renal	1.00 (0.02)	0.43 (0.13, 0.71)	-0.05 (-61.92, 61.53)

Table E37: Mixture probability estimates (as mean and standard deviation), and informative and vague mixture component estimates (as median and 95% credible interval), for conditional variances from application of surrogacy models assuming equal mixture probabilities across surrogacy parameters to data at present-day. MCIP - Mixed common and independent parameters, MRIP - Mixed random and independent parameters, NSCLC - Non-small cell lung cancer, OFTPP - Ovarian, fallopian tube, and primary peritoneal.

Model	Indication	Probability	Informative	Vague
MCIP	Breast	1.00 (0.00)	0.05 (0.00, 0.11)	0.34 (0.02, 1.11)
MCIP	Cervical	0.99 (0.08)	0.05 (0.00, 0.11)	0.34 (0.02, 1.12)
MCIP	Colorectal	1.00 (0.00)	0.05 (0.00, 0.11)	0.34 (0.02, 1.14)
MCIP	Glioblastoma	1.00 (0.03)	0.05 (0.00, 0.11)	0.34 (0.02, 1.12)
MCIP	NSCLC	1.00 (0.00)	0.05 (0.00, 0.11)	0.34 (0.02, 1.12)
MCIP	OFTPP	1.00 (0.00)	0.05 (0.00, 0.11)	0.34 (0.02, 1.12)
MCIP	Renal	1.00 (0.01)	0.05 (0.00, 0.11)	0.34 (0.02, 1.12)
MRIP	Breast	1.00 (0.00)	0.04 (0.00, 0.13)	0.34 (0.02, 1.12)
MRIP	Cervical	0.99 (0.09)	0.05 (0.00, 0.20)	0.34 (0.02, 1.12)
MRIP	Colorectal	1.00 (0.00)	0.04 (0.00, 0.11)	0.34 (0.02, 1.12)
MRIP	Glioblastoma	1.00 (0.01)	0.05 (0.00, 0.18)	0.34 (0.02, 1.12)
MRIP	NSCLC	1.00 (0.00)	0.04 (0.00, 0.14)	0.34 (0.02, 1.12)
MRIP	OFTPP	1.00 (0.00)	0.04 (0.00, 0.14)	0.34 (0.02, 1.12)
MRIP	Renal	1.00 (0.02)	0.04 (0.00, 0.15)	0.34 (0.02, 1.12)

E.6 Sensitivity analysis: independent conditional variance parameters

E.6.1 Surrogacy parameter estimates

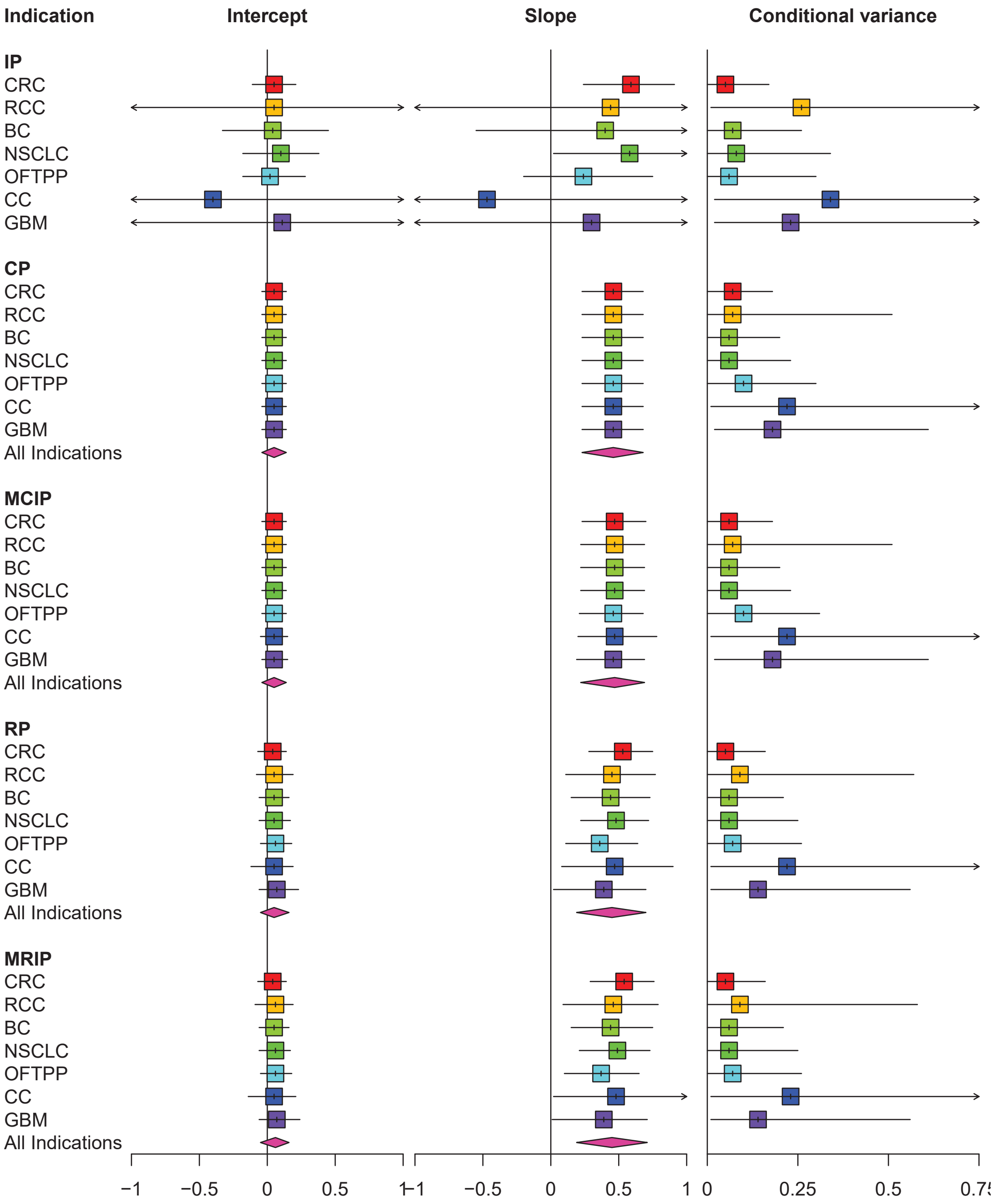

Figure E2: Multi-indication meta-analysis of surrogacy parameters, assuming independent conditional variance parameters, using data available at present day. IP - independent parameters, CP - common parameter, MCIP - mixed common and independent parameters, RP - random parameters, MRIP - mixed random and independent parameters, CRC - colorectal cancer, BC - breast cancer, NSCLC - non-small cell lung cancer, RCC - renal cell carcinoma, CC - cervical cancer, GBM - glioblastoma, OFTPP - ovarian, fallopian tube, and primary peritoneal.

Table E38: Median and 95% credible interval estimates for intercepts from surrogacy models assuming independent conditional variances, using data available at present-day. IP - Independent parameters, CP - Common parameters, MCIP - Mixed common and independent parameters, RP - Random parameters, MRIP - Mixed random and independent parameters, BC - Breast cancer, CC - Cervical cancer, CRC - Colorectal cancer, GBM - Glioblastoma, NSCLC - Non-small cell lung cancer, OFTPP - Ovarian, fallopian tube, and primary peritoneal, RCC - Renal cell carcinoma.

Indication	IP	CP	MCIP	RP	MRIP
CRC	0.05 (-0.11, 0.21)	0.05 (-0.04, 0.14)	0.05 (-0.04, 0.14)	0.04 (-0.07, 0.14)	0.04 (-0.07, 0.14)
RCC	0.05 (-12.75, 15.19)	0.05 (-0.04, 0.14)	0.05 (-0.04, 0.14)	0.05 (-0.08, 0.19)	0.06 (-0.09, 0.19)
BC	0.04 (-0.33, 0.45)	0.05 (-0.04, 0.14)	0.05 (-0.04, 0.14)	0.05 (-0.06, 0.16)	0.05 (-0.06, 0.16)
NSCLC	0.10 (-0.18, 0.38)	0.05 (-0.04, 0.14)	0.05 (-0.04, 0.14)	0.05 (-0.06, 0.17)	0.06 (-0.06, 0.17)
OFTPP	0.02 (-0.18, 0.28)	0.05 (-0.04, 0.14)	0.05 (-0.04, 0.14)	0.06 (-0.05, 0.18)	0.06 (-0.05, 0.18)
CC	-0.40 (-22.89, 23.86)	0.05 (-0.04, 0.14)	0.05 (-0.05, 0.15)	0.05 (-0.12, 0.19)	0.05 (-0.14, 0.21)
GBM	0.11 (-1.52, 1.64)	0.05 (-0.04, 0.14)	0.05 (-0.04, 0.15)	0.07 (-0.06, 0.23)	0.07 (-0.06, 0.24)
All Indications		0.05 (-0.04, 0.14)	0.05 (-0.04, 0.14)	0.05 (-0.05, 0.16)	0.06 (-0.05, 0.16)

Table E39: Median and 95% credible interval estimates for slopes from surrogacy models assuming independent conditional variances, using data available at present-day. IP - Independent parameters, CP - Common parameters, MCIP - Mixed common and independent parameters, RP - Random parameters, MRIP - Mixed random and independent parameters, BC - Breast cancer, CC - Cervical cancer, CRC - Colorectal cancer, GBM - Glioblastoma, NSCLC - Non-small cell lung cancer, OFTPP - Ovarian, fallopian tube, and primary peritoneal, RCC - Renal cell carcinoma.

Indication	IP	CP	MCIP	RP	MRIP
CRC	0.59 (0.24, 0.91)	0.46 (0.23, 0.68)	0.47 (0.23, 0.70)	0.53 (0.28, 0.75)	0.54 (0.29, 0.76)
RCC	0.44 (-28.08, 34.37)	0.46 (0.23, 0.68)	0.47 (0.22, 0.69)	0.45 (0.11, 0.77)	0.46 (0.09, 0.79)
BC	0.40 (-0.55, 1.60)	0.46 (0.23, 0.68)	0.47 (0.22, 0.69)	0.44 (0.15, 0.73)	0.44 (0.15, 0.75)
NSCLC	0.58 (0.02, 1.12)	0.46 (0.23, 0.68)	0.47 (0.22, 0.69)	0.48 (0.22, 0.72)	0.49 (0.21, 0.73)
OFTPP	0.24 (-0.20, 0.75)	0.46 (0.23, 0.68)	0.46 (0.21, 0.68)	0.36 (0.11, 0.64)	0.37 (0.10, 0.65)
CC	-0.47 (-54.65, 59.38)	0.46 (0.23, 0.68)	0.47 (0.20, 0.78)	0.47 (0.08, 0.90)	0.48 (0.02, 1.11)
GBM	0.30 (-3.14, 3.47)	0.46 (0.23, 0.68)	0.46 (0.19, 0.69)	0.39 (0.02, 0.70)	0.39 (0.01, 0.71)
All Indications		0.46 (0.23, 0.68)	0.47 (0.22, 0.69)	0.45 (0.19, 0.70)	0.45 (0.19, 0.71)

Table E40: Median and 95% credible interval estimates for conditonal variances from surrogacy models assuming independent conditional variances, using data available at present-day. IP - Independent parameters, CP - Common parameters, MCIP - Mixed common and independent parameters, RP - Random parameters, MRIP - Mixed random and independent parameters, BC - Breast cancer, CC - Cervical cancer, CRC - Colorectal cancer, GBM - Glioblastoma, NSCLC - Non-small cell lung cancer, OFTPP - Ovarian, fallopian tube, and primary peritoneal, RCC - Renal cell carcinoma.

Indication	IP	CP	MCIP	RP	MRIP
CRC	0.05 (0.00, 0.17)	0.07 (0.00, 0.18)	0.06 (0.00, 0.18)	0.05 (0.00, 0.16)	0.05 (0.00, 0.16)
RCC	0.26 (0.01, 1.02)	0.07 (0.00, 0.51)	0.07 (0.00, 0.51)	0.09 (0.00, 0.57)	0.09 (0.00, 0.58)
BC	0.07 (0.00, 0.26)	0.06 (0.00, 0.20)	0.06 (0.00, 0.20)	0.06 (0.00, 0.21)	0.06 (0.00, 0.21)
NSCLC	0.08 (0.00, 0.34)	0.06 (0.00, 0.23)	0.06 (0.00, 0.23)	0.06 (0.00, 0.25)	0.06 (0.00, 0.25)
OFTPP	0.06 (0.00, 0.30)	0.10 (0.00, 0.30)	0.10 (0.00, 0.31)	0.07 (0.00, 0.26)	0.07 (0.00, 0.26)
CC	0.34 (0.02, 1.12)	0.22 (0.01, 0.90)	0.22 (0.01, 0.92)	0.22 (0.01, 0.91)	0.23 (0.01, 0.92)
GBM	0.23 (0.02, 0.91)	0.18 (0.02, 0.61)	0.18 (0.02, 0.61)	0.14 (0.01, 0.56)	0.14 (0.01, 0.56)

E.6.2 Deviance information criterion (DIC)

Table E41: Residual deviance, number of effective parameters, and deviance information criterian (DIC) from application of surrogacy models assuming independent condition variances to data at present-day. *Based on 38 data points. IP - Independent parameters, CP - Common parameter, MCIP - Mixed common and independent parameters, RP - Random parameters, MRIP - Mixed random and independent parameters

Model	Residual deviance	Deviance	p_D	DIC
IP	70.95	-127.59	48.75	-78.84
CP	71.62	-125.80	44.36	-81.44
MCIP	71.49	-125.88	45.01	-80.86
RP	71.36	-128.82	45.04	-83.79
MRIP	71.41	-128.01	45.36	-82.65

E.6.3 Mixture probabilities

Table E42: Mixture probability estimates (as mean and standard deviation), and informative and vague mixture component estimates (as median and 95% credible interval), for intercepts from application of surrogacy models assuming independent conditional variances to data at present-day. MCIP - Mixed common and independent parameters, MRIP - Mixed random and independent parameters, NSCLC - Non-small cell lung cancer, OFTPP - Ovarian, fallopian tube, and primary peritoneal.

Model	Indication	Probability	Informative	Vague
MCIP	Breast	1.00 (0.03)	0.05 (-0.04, 0.14)	0.09 (-61.55, 62.29)
MCIP	Cervical	0.98 (0.14)	0.05 (-0.04, 0.14)	0.03 (-61.70, 61.62)
MCIP	Colorectal	0.99 (0.08)	0.05 (-0.04, 0.14)	-0.01 (-62.29, 61.80)
MCIP	Glioblastoma	0.99 (0.10)	0.05 (-0.04, 0.14)	0.15 (-62.04, 61.75)
MCIP	NSCLC	1.00 (0.05)	0.05 (-0.04, 0.14)	0.03 (-62.26, 62.03)
MCIP	OFTPP	1.00 (0.07)	0.05 (-0.04, 0.14)	0.09 (-61.54, 62.38)
MCIP	Renal	1.00 (0.06)	0.05 (-0.04, 0.14)	-0.03 (-61.98, 62.25)
MRIP	Breast	1.00 (0.05)	0.05 (-0.06, 0.16)	-0.19 (-62.06, 61.65)
MRIP	Cervical	0.98 (0.14)	0.05 (-0.12, 0.20)	0.05 (-61.73, 61.71)
MRIP	Colorectal	1.00 (0.07)	0.04 (-0.07, 0.14)	0.05 (-61.91, 61.80)
MRIP	Glioblastoma	0.99 (0.09)	0.07 (-0.06, 0.23)	0.13 (-61.92, 61.71)
MRIP	NSCLC	1.00 (0.05)	0.06 (-0.06, 0.17)	0.06 (-62.08, 61.94)
MRIP	OFTPP	1.00 (0.06)	0.06 (-0.05, 0.18)	0.01 (-61.93, 62.03)
MRIP	Renal	0.99 (0.09)	0.06 (-0.09, 0.19)	-0.04 (-61.95, 61.60)

Table E43: Mixture probability estimates (as mean and standard deviation), and informative and vague mixture component estimates (as median and 95% credible interval), for slopes from application of surrogacy models assuming independent conditional variances to data at present-day. MCIP - Mixed common and independent parameters, MRIP - Mixed random and independent parameters, NSCLC - Non-small cell lung cancer, OFTPP - Ovarian, fallopian tube, and primary peritoneal.

Model	Indication	Probability	Informative	Vague
MCIP	Breast	1.00 (0.06)	0.47 (0.22, 0.69)	0.22 (-61.85, 61.96)
MCIP	Cervical	0.96 (0.20)	0.47 (0.22, 0.69)	0.55 (-61.49, 61.38)
MCIP	Colorectal	0.97 (0.17)	0.47 (0.22, 0.69)	0.52 (-61.69, 61.95)
MCIP	Glioblastoma	0.97 (0.16)	0.47 (0.22, 0.69)	0.16 (-61.87, 61.65)
MCIP	NSCLC	0.99 (0.07)	0.47 (0.22, 0.69)	0.21 (-61.93, 61.86)
MCIP	OFTPP	0.97 (0.16)	0.47 (0.22, 0.69)	0.26 (-61.49, 61.18)
MCIP	Renal	0.99 (0.09)	0.47 (0.22, 0.69)	0.21 (-61.89, 61.97)
MRIP	Breast	0.99 (0.09)	0.44 (0.15, 0.75)	0.23 (-61.95, 61.83)
MRIP	Cervical	0.96 (0.20)	0.48 (0.07, 0.92)	0.52 (-61.27, 61.66)
MRIP	Colorectal	0.99 (0.11)	0.54 (0.28, 0.76)	0.37 (-62.10, 61.72)
MRIP	Glioblastoma	0.98 (0.13)	0.39 (0.02, 0.71)	0.13 (-61.26, 61.56)
MRIP	NSCLC	0.99 (0.08)	0.49 (0.21, 0.73)	0.22 (-62.03, 61.99)
MRIP	OFTPP	0.99 (0.11)	0.37 (0.10, 0.66)	0.23 (-61.87, 61.56)
MRIP	Renal	0.99 (0.12)	0.46 (0.10, 0.78)	0.14 (-61.95, 61.78)

E.7 Sensitivity analysis: excluding data on breast cancer

E.7.1 Surrogacy parameter estimates

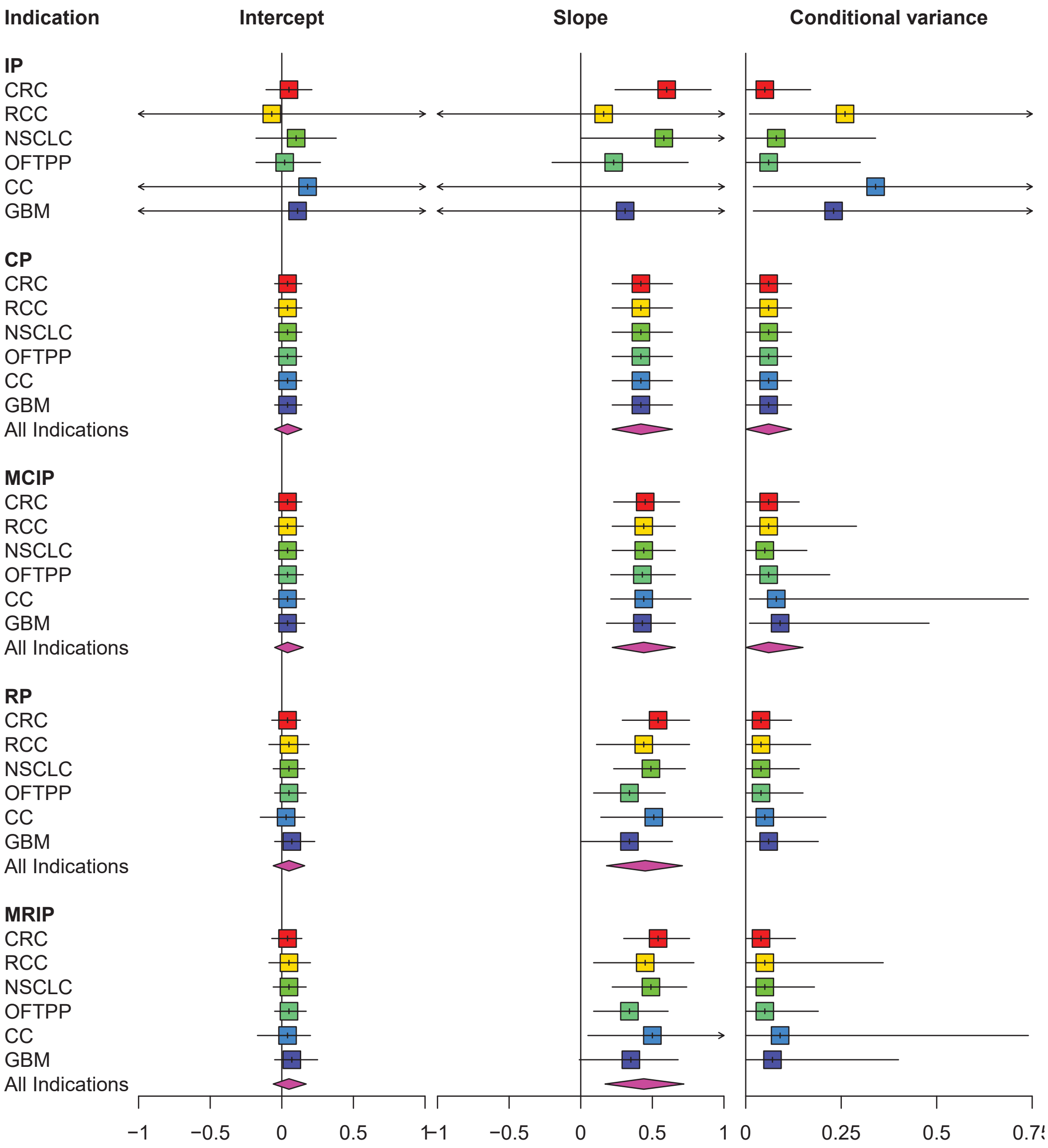

Figure E3: Multi-indication meta-analysis of surrogacy parameters using data available at present day, excluding data from trials in breast cancer. IP - independent parameters, CP - common parameter, MCIP - mixed common and independent parameters, RP - random parameters, MRIP - mixed random and independent parameters, CRC - colorectal cancer, BC - breast cancer, NSCLC - non-small cell lung cancer, RCC - renal cell carcinoma, CC - cervical cancer, GBM - glioblastoma, OFTPP - ovarian, fallopian tube, and primary peritoneal.

Table E44: Median and 95% credible interval estimates for intercepts from surrogacy models, using data available at present-day excluding breast cancer. IP - Independent parameters, CP - Common parameters, MCIP - Mixed common and independent parameters, RP - Random parameters, MRIP - Mixed random and independent parameters, CC - Cervical cancer, CRC - Colorectal cancer, GBM - Glioblastoma, NSCLC - Non-small cell lung cancer, OFTPP - Ovarian, fallopian tube, and primary peritoneal, RCC - Renal cell carcinoma.

Indication	IP	CP	MCIP	RP	MRIP
CRC	0.05 (-0.11, 0.21)	0.04 (-0.05, 0.14)	0.04 (-0.05, 0.14)	0.04 (-0.07, 0.13)	0.04 (-0.07, 0.14)
RCC	-0.07 (-15.57, 14.90)	0.04 (-0.05, 0.14)	0.04 (-0.05, 0.15)	0.05 (-0.09, 0.19)	0.05 (-0.09, 0.20)
NSCLC	0.10 (-0.18, 0.38)	0.04 (-0.05, 0.14)	0.04 (-0.05, 0.15)	0.05 (-0.06, 0.16)	0.05 (-0.06, 0.17)
OFTPP	0.02 (-0.18, 0.27)	0.04 (-0.05, 0.14)	0.04 (-0.05, 0.15)	0.05 (-0.05, 0.17)	0.05 (-0.05, 0.17)
CC	0.18 (-22.65, 23.18)	0.04 (-0.05, 0.14)	0.04 (-0.06, 0.16)	0.03 (-0.15, 0.16)	0.04 (-0.17, 0.20)
GBM	0.11 (-1.59, 1.79)	0.04 (-0.05, 0.14)	0.04 (-0.05, 0.16)	0.07 (-0.05, 0.23)	0.07 (-0.05, 0.25)
All Indications		0.04 (-0.05, 0.14)	0.04 (-0.05, 0.15)	0.05 (-0.06, 0.16)	0.05 (-0.06, 0.17)

Table E45: Median and 95% credible interval estimates for slopes from surrogacy models, using data available at present-day excluding breast cancer. IP - Independent parameters, CP - Common parameters, MCIP - Mixed common and independent parameters, RP - Random parameters, MRIP - Mixed random and independent parameters, CC - Cervical cancer, CRC - Colorectal cancer, GBM - Glioblastoma, NSCLC - Non-small cell lung cancer, OFTPP - Ovarian, fallopian tube, and primary peritoneal, RCC - Renal cell carcinoma.

Indication	IP	CP	MCIP	RP	MRIP
CRC	0.60 (0.24, 0.91)	0.42 (0.22, 0.64)	0.45 (0.23, 0.69)	0.54 (0.29, 0.76)	0.54 (0.30, 0.76)
RCC	0.16 (-34.92, 34.43)	0.42 (0.22, 0.64)	0.44 (0.22, 0.66)	0.44 (0.11, 0.76)	0.45 (0.09, 0.79)
NSCLC	0.58 (0.00, 1.13)	0.42 (0.22, 0.64)	0.44 (0.22, 0.66)	0.49 (0.23, 0.73)	0.49 (0.22, 0.74)
OFTPP	0.23 (-0.20, 0.75)	0.42 (0.22, 0.64)	0.43 (0.21, 0.66)	0.34 (0.09, 0.59)	0.34 (0.09, 0.61)
CC	1.23 (-56.06, 57.32)	0.42 (0.22, 0.64)	0.44 (0.21, 0.77)	0.51 (0.14, 0.99)	0.50 (0.05, 1.08)
GBM	0.31 (-3.32, 3.79)	0.42 (0.22, 0.64)	0.43 (0.18, 0.66)	0.34 (0.00, 0.64)	0.35 (-0.01, 0.68)
All Indications		0.42 (0.22, 0.64)	0.44 (0.22, 0.66)	0.45 (0.18, 0.71)	0.44 (0.17, 0.72)

Table E46: Median and 95% credible interval estimates for conditonal variances from surrogacy models, using data available at present-day excluding breast cancer. IP - Independent parameters, CP - Common parameters, MCIP - Mixed common and independent parameters, RP - Random parameters, MRIP - Mixed random and independent parameters, CC - Cervical cancer, CRC - Colorectal cancer, GBM - Glioblastoma, NSCLC - Non-small cell lung cancer, OFTPP - Ovarian, fallopian tube, and primary peritoneal, RCC - Renal cell carcinoma.

Indication	IP	CP	MCIP	RP	MRIP
CRC	0.05 (0.00, 0.17)	0.06 (0.00, 0.12)	0.06 (0.00, 0.14)	0.04 (0.00, 0.12)	0.04 (0.00, 0.13)
RCC	0.26 (0.01, 1.01)	0.06 (0.00, 0.12)	0.06 (0.00, 0.29)	0.04 (0.00, 0.17)	0.05 (0.00, 0.36)
NSCLC	0.08 (0.00, 0.34)	0.06 (0.00, 0.12)	0.05 (0.00, 0.16)	0.04 (0.00, 0.14)	0.05 (0.00, 0.18)
OFTPP	0.06 (0.00, 0.30)	0.06 (0.00, 0.12)	0.06 (0.00, 0.22)	0.04 (0.00, 0.15)	0.05 (0.00, 0.19)
CC	0.34 (0.02, 1.12)	0.06 (0.00, 0.12)	0.08 (0.01, 0.74)	0.05 (0.00, 0.21)	0.09 (0.00, 0.74)
GBM	0.23 (0.02, 0.92)	0.06 (0.00, 0.12)	0.09 (0.01, 0.48)	0.06 (0.00, 0.19)	0.07 (0.00, 0.40)
All Indications		0.06 (0.00, 0.12)	0.06 (0.00, 0.15)		

E.7.2 Deviance information criterion (DIC)

Table E47: Residual deviance, number of effective parameters, and deviance information criterian (DIC) from application of surrogacy models to data at present-day excluding breast cancer. *Based on 28 data points. IP - Independent parameters, CP - Common parameters, MCIP - Mixed common and independent parameters, RP - Random parameters, MRIP - Mixed random and independent parameters.

Model	Residual deviance	Deviance	p_D	DIC
IP	51.28	-99.09	36.04	-63.05
CP	52.71	-93.98	30.04	-63.94
MCIP	52.37	-90.34	31.71	-58.63
RP	51.43	-96.24	32.16	-64.08
MRIP	51.02	-88.42	32.97	-55.45

E.7.3 Mixture probabilities

Table E48: Mixture probability estimates (as mean and standard deviation), and informative and vague mixture component estimates (as median and 95% credible interval), for intercepts from application of surrogacy models to data at present-day excluding breast cancer. MCIP - Mixed common and independent parameters, MRIP - Mixed random and independent parameters, NSCLC - Non-small cell lung cancer, OFTPP - Ovarian, fallopian tube, and primary peritoneal.

Model	Indication	Probability	Informative	Vague
MCIP	Cervical	0.98 (0.13)	0.04 (-0.05, 0.15)	0.03 (-61.60, 61.94)
MCIP	Colorectal	0.99 (0.08)	0.04 (-0.05, 0.15)	-0.01 (-61.56, 62.32)
MCIP	Glioblastoma	0.98 (0.14)	0.04 (-0.05, 0.15)	0.15 (-61.27, 61.81)
MCIP	NSCLC	1.00 (0.04)	0.04 (-0.05, 0.15)	0.07 (-62.34, 62.44)
MCIP	OFTPP	1.00 (0.07)	0.04 (-0.05, 0.15)	-0.02 (-62.18, 61.70)
MCIP	Renal	1.00 (0.05)	0.04 (-0.05, 0.15)	-0.04 (-61.93, 61.91)
MRIP	Cervical	0.98 (0.13)	0.04 (-0.15, 0.19)	-0.08 (-61.53, 61.48)
MRIP	Colorectal	1.00 (0.07)	0.04 (-0.07, 0.14)	-0.01 (-61.86, 61.92)
MRIP	Glioblastoma	0.99 (0.09)	0.07 (-0.06, 0.25)	0.16 (-61.84, 62.11)
MRIP	NSCLC	1.00 (0.06)	0.05 (-0.06, 0.17)	0.05 (-62.30, 61.86)
MRIP	OFTPP	1.00 (0.05)	0.05 (-0.05, 0.17)	-0.06 (-62.03, 62.23)
MRIP	Renal	0.99 (0.07)	0.05 (-0.09, 0.20)	-0.04 (-62.49, 61.87)

Table E49: Mixture probability estimates (as mean and standard deviation), and informative and vague mixture component estimates (as median and 95% credible interval), for slopes from application of surrogacy models to data at present-day excluding breast cancer. MCIP - Mixed common and independent parameters, MRIP - Mixed random and independent parameters, NSCLC - Non-small cell lung cancer, OFTPP - Ovarian, fallopian tube, and primary peritoneal.

Model	Indication	Probability	Informative	Vague
MCIP	Cervical	0.96 (0.19)	0.44 (0.22, 0.66)	0.67 (-61.30, 61.40)
MCIP	Colorectal	0.95 (0.21)	0.44 (0.22, 0.66)	0.59 (-61.19, 61.35)
MCIP	Glioblastoma	0.97 (0.17)	0.44 (0.22, 0.66)	0.15 (-61.70, 61.26)
MCIP	NSCLC	1.00 (0.06)	0.44 (0.22, 0.66)	0.16 (-61.63, 61.99)
MCIP	OFTPP	0.97 (0.17)	0.44 (0.22, 0.66)	0.25 (-61.77, 61.38)
MCIP	Renal	0.99 (0.08)	0.44 (0.22, 0.66)	0.25 (-61.80, 61.69)
MRIP	Cervical	0.97 (0.18)	0.49 (0.07, 1.00)	0.52 (-61.66, 61.54)
MRIP	Colorectal	0.99 (0.11)	0.54 (0.29, 0.76)	0.46 (-61.90, 61.58)
MRIP	Glioblastoma	0.98 (0.13)	0.36 (0.00, 0.68)	0.16 (-61.65, 62.05)
MRIP	NSCLC	0.99 (0.09)	0.49 (0.22, 0.74)	0.32 (-62.12, 61.84)
MRIP	OFTPP	0.99 (0.12)	0.34 (0.09, 0.62)	0.25 (-61.98, 61.69)
MRIP	Renal	0.99 (0.10)	0.45 (0.10, 0.79)	0.34 (-61.93, 61.54)

E.8 Sensitivity analysis: common effect across trials within each indication

E.8.1 Treatment effect estimates

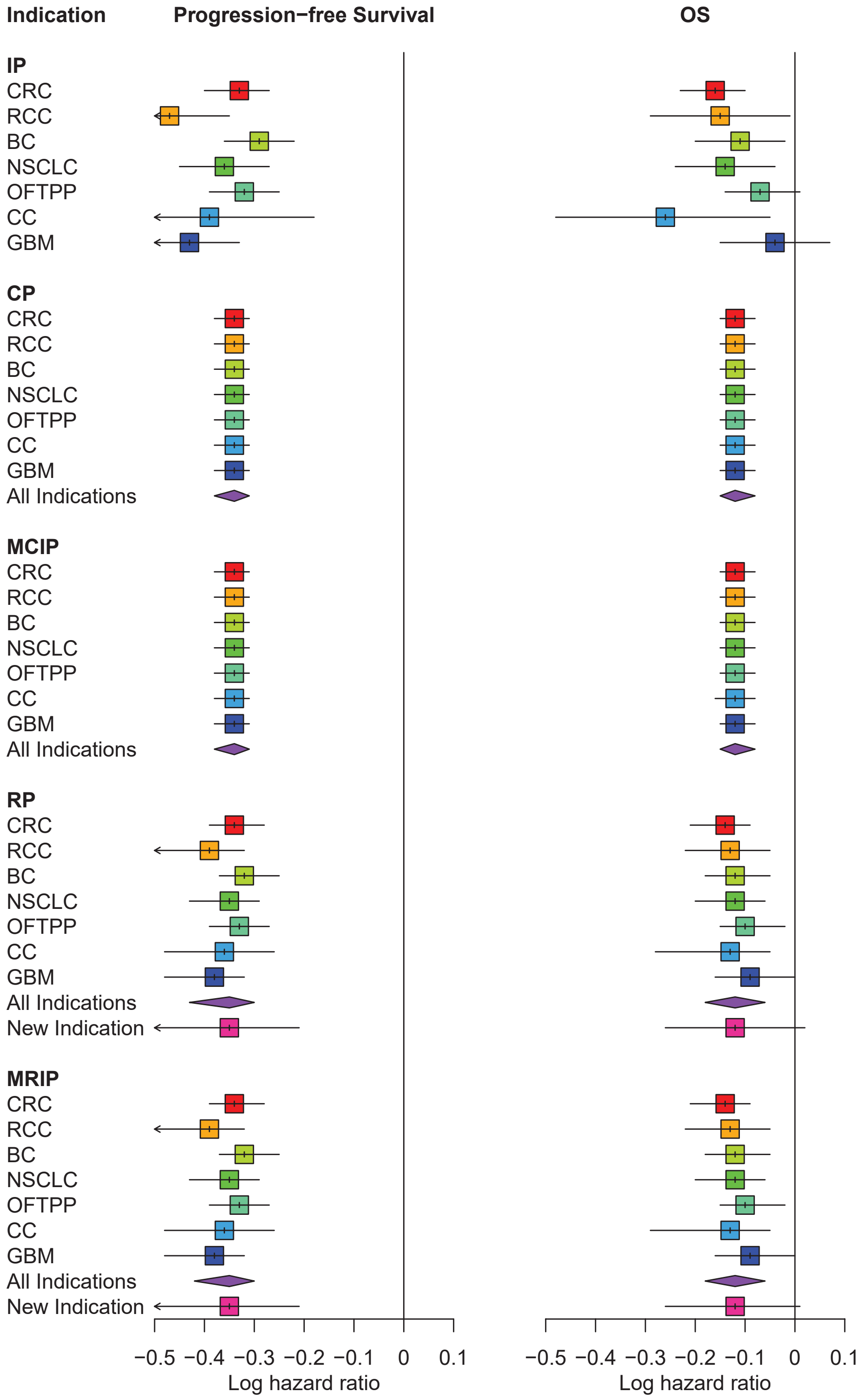

Figure E4: Multi-indication meta-analysis, assuming a common effect across trials within each indication, synthesising effects on overall survival (OS) and progression-free survival (PFS) using data available at present day. IP - independent parameters, CP - common parameter, MCIP - mixed common and independent parameters, RP - random parameters, MRIP - mixed random and independent parameters, CRC - colorectal cancer, BC - breast cancer, NSCLC - non-small cell lung cancer, RCC - renal cell carcinoma, CC - cervical cancer, GBM - glioblastoma, OFTPP - ovarian, fallopian tube, and primary peritoneal.

Table E50: Median and 95% credible interval estimates for hazard ratios from multi-indication meta-analysis models, assuming a common effect across trials within each indication, using data on overall survival (OS) available at present-day. IP - Independent parameters, CP - Common parameters, MCIP - Mixed common and independent parameters, RP - Random parameters, MRIP - Mixed random and independent parameters, BC - Breast cancer, CC - Cervical cancer, CRC - Colorectal cancer, GBM - Glioblastoma, NSCLC - Non-small cell lung cancer, OFTPP - Ovarian, fallopian tube, and primary peritoneal, RCC - Renal cell carcinoma.

Indication	IP	CP	MCIP	RP	MRIP
CRC	0.85 (0.79, 0.90)	0.89 (0.86, 0.92)	0.89 (0.86, 0.92)	0.87 (0.81, 0.91)	0.87 (0.81, 0.91)
RCC	0.86 (0.75, 0.99)	0.89 (0.86, 0.92)	0.89 (0.86, 0.92)	0.88 (0.80, 0.95)	0.88 (0.80, 0.95)
BC	0.90 (0.82, 0.98)	0.89 (0.86, 0.92)	0.89 (0.86, 0.92)	0.89 (0.84, 0.95)	0.89 (0.84, 0.95)
NSCLC	0.87 (0.79, 0.96)	0.89 (0.86, 0.92)	0.89 (0.86, 0.92)	0.89 (0.82, 0.94)	0.89 (0.82, 0.94)
OFTPP	0.93 (0.87, 1.01)	0.89 (0.86, 0.92)	0.89 (0.86, 0.92)	0.90 (0.86, 0.98)	0.90 (0.86, 0.98)
CC	0.77 (0.62, 0.95)	0.89 (0.86, 0.92)	0.89 (0.85, 0.92)	0.88 (0.76, 0.95)	0.88 (0.75, 0.95)
GBM	0.96 (0.86, 1.07)	0.89 (0.86, 0.92)	0.89 (0.86, 0.92)	0.91 (0.85, 1.00)	0.91 (0.85, 1.00)
All Indications		0.89 (0.86, 0.92)	0.89 (0.86, 0.92)	0.89 (0.84, 0.94)	0.89 (0.84, 0.94)
New Indication				0.89 (0.77, 1.02)	0.89 (0.77, 1.01)

Table E51: Median and 95% credible interval estimates for hazard ratios from multi-indication meta-analysis models, assuming a common effect across trials within each indication, using data on progression-free survival (PFS) available at present-day. IP - Independent parameters, CP - Common parameters, MCIP - Mixed common and independent parameters, RP - Random parameters, MRIP - Mixed random and independent parameters, BC - Breast cancer, CC - Cervical cancer, CRC - Colorectal cancer, GBM - Glioblastoma, NSCLC - Non-small cell lung cancer, OFTPP - Ovarian, fallopian tube, and primary peritoneal, RCC - Renal cell carcinoma.

Indication	IP	CP	MCIP	RP	MRIP
CRC	0.72 (0.67, 0.76)	0.71 (0.68, 0.73)	0.71 (0.68, 0.73)	0.71 (0.68, 0.76)	0.71 (0.68, 0.76)
RCC	0.63 (0.56, 0.70)	0.71 (0.68, 0.73)	0.71 (0.68, 0.73)	0.68 (0.60, 0.73)	0.68 (0.60, 0.73)
BC	0.75 (0.70, 0.80)	0.71 (0.68, 0.73)	0.71 (0.68, 0.73)	0.73 (0.69, 0.78)	0.73 (0.69, 0.78)
NSCLC	0.70 (0.64, 0.76)	0.71 (0.68, 0.73)	0.71 (0.68, 0.73)	0.70 (0.65, 0.75)	0.70 (0.65, 0.75)
OFTPP	0.73 (0.68, 0.78)	0.71 (0.68, 0.73)	0.71 (0.68, 0.73)	0.72 (0.68, 0.76)	0.72 (0.68, 0.76)
CC	0.68 (0.55, 0.84)	0.71 (0.68, 0.73)	0.71 (0.68, 0.73)	0.70 (0.62, 0.77)	0.70 (0.62, 0.77)
GBM	0.65 (0.59, 0.72)	0.71 (0.68, 0.73)	0.71 (0.68, 0.73)	0.68 (0.62, 0.73)	0.68 (0.62, 0.73)
All Indications		0.71 (0.68, 0.73)	0.71 (0.68, 0.73)	0.70 (0.65, 0.74)	0.70 (0.66, 0.74)
New Indication				0.70 (0.59, 0.81)	0.70 (0.59, 0.81)

E.8.2 Deviance information criterion (DIC)

Table E52: Residual deviance, deviance, number of effective parameters, and deviance information criterian (DIC) from application of models assuming a common effect within indications to data on overall survival at present-day. *Based on 38 data points. IP - Independent parameters, CP - Common parameter, MCIP - Mixed common and independent parameters, RP - Random parameters, MRIP - Mixed random and independent parameters

Model	Residual deviance	Deviance	p_D	DIC
IP	48.25	-34.07	7.01	-27.06
CP	50.19	-32.13	1.01	-31.12
MCIP	50.17	-32.16	1.05	-31.10
RP	48.14	-34.19	3.75	-30.44
MRIP	48.13	-32.81	4.05	-28.76

Table E53: Residual deviance, deviance, number of effective parameters, and deviance information criterian (DIC) from application of models assuming a common effect within indications to data on progression-free survival at present-day. *Based on 43 data points. IP - Independent parameters, CP - Common parameter, MCIP - Mixed common and independent parameters, RP - Random parameters, MRIP - Mixed random and independent parameters

Model	Residual deviance	Deviance	p_D	DIC
IP	147.07	50.13	7.01	57.14
CP	151.29	54.34	1.01	55.35
MCIP	151.21	54.26	1.17	55.43
RP	147.42	50.47	4.53	55.00
MRIP	147.42	51.86	5.80	57.67

E.8.3 Mixture probabilities

Table E54: Mixture probability estimates (as mean and standard deviation), and informative and vague mixture component estimates (as median and 95% credible interval), from application of models, assuming a common effect across trials within each indication, to data on overall survival at present-day. IP - Independent parameters, CP - Common parameter, MCIP - Mixed common and independent parameters, RP - Random parameters, MRIP - Mixed random and independent parameters

Model	Indication	Probability	Informative	Vague
MCIP	Breast	1.00 (0.04)	-0.12 (-0.15, -0.08)	-0.12 (-62.11, 62.07)
MCIP	Cervical	0.99 (0.09)	-0.12 (-0.15, -0.08)	-0.17 (-62.03, 62.29)
MCIP	Colorectal	1.00 (0.07)	-0.12 (-0.15, -0.08)	-0.15 (-61.93, 61.76)
MCIP	Glioblastoma	0.99 (0.08)	-0.12 (-0.15, -0.08)	-0.03 (-61.57, 61.68)
MCIP	NSCLC	1.00 (0.04)	-0.12 (-0.15, -0.08)	-0.07 (-61.64, 62.05)
MCIP	OFTPP	1.00 (0.07)	-0.12 (-0.15, -0.08)	-0.06 (-61.83, 61.58)
MCIP	Renal	1.00 (0.05)	-0.12 (-0.15, -0.08)	-0.09 (-61.78, 61.72)
MRIP	Breast	1.00 (0.05)	-0.12 (-0.18, -0.05)	-0.04 (-61.92, 61.70)
MRIP	Cervical	0.99 (0.09)	-0.13 (-0.28, -0.05)	-0.18 (-61.90, 61.86)
MRIP	Colorectal	1.00 (0.06)	-0.14 (-0.21, -0.09)	-0.13 (-61.97, 61.74)
MRIP	Glioblastoma	0.99 (0.08)	-0.09 (-0.16, 0.00)	-0.04 (-61.91, 61.88)
MRIP	NSCLC	1.00 (0.06)	-0.12 (-0.20, -0.06)	-0.13 (-62.00, 61.88)
MRIP	OFTPP	1.00 (0.07)	-0.10 (-0.15, -0.02)	-0.06 (-62.25, 62.27)
MRIP	Renal	1.00 (0.06)	-0.13 (-0.22, -0.05)	-0.08 (-62.22, 62.02)

Table E55: Mixture probability estimates (as mean and standard deviation), and informative and vague mixture component estimates (as median and 95% credible interval), from application of models, assuming a common effect across trials within each indication, to data on progression-free survival at present-day. IP - Independent parameters, CP - Common parameter, MCIP - Mixed common and independent parameters, RP - Random parameters, MRIP - Mixed random and independent parameters

Model	Indication	Probability	Informative	Vague
MCIP	Breast	0.99 (0.07)	-0.34 (-0.38, -0.31)	-0.26 (-62.12, 61.94)
MCIP	Cervical	1.00 (0.06)	-0.34 (-0.38, -0.31)	-0.12 (-62.10, 62.26)
MCIP	Colorectal	1.00 (0.03)	-0.34 (-0.38, -0.31)	-0.10 (-62.08, 61.76)
MCIP	Glioblastoma	0.99 (0.09)	-0.34 (-0.38, -0.31)	-0.23 (-61.49, 61.68)
MCIP	NSCLC	1.00 (0.04)	-0.34 (-0.38, -0.31)	-0.08 (-61.71, 61.95)
MCIP	OFTPP	1.00 (0.04)	-0.34 (-0.38, -0.31)	-0.08 (-62.05, 61.53)
MCIP	Renal	0.98 (0.13)	-0.34 (-0.38, -0.31)	-0.42 (-61.55, 61.55)
MRIP	Breast	1.00 (0.07)	-0.32 (-0.37, -0.25)	-0.18 (-61.98, 61.73)
MRIP	Cervical	1.00 (0.06)	-0.36 (-0.48, -0.26)	-0.16 (-61.85, 61.57)
MRIP	Colorectal	1.00 (0.05)	-0.34 (-0.39, -0.28)	-0.26 (-62.08, 61.48)
MRIP	Glioblastoma	0.99 (0.07)	-0.38 (-0.48, -0.31)	-0.27 (-62.26, 61.80)
MRIP	NSCLC	1.00 (0.06)	-0.35 (-0.43, -0.29)	-0.21 (-62.26, 61.88)
MRIP	OFTPP	1.00 (0.05)	-0.33 (-0.39, -0.27)	-0.10 (-61.68, 62.23)
MRIP	Renal	0.99 (0.11)	-0.39 (-0.51, -0.31)	-0.29 (-61.66, 62.06)
